# Submaximal Fitness Tests in Team Sports: A Theoretical Framework for Evaluating Physiological State

**DOI:** 10.1007/s40279-022-01712-0

**Published:** 2022-07-11

**Authors:** Tzlil Shushan, Shaun J. McLaren, Martin Buchheit, Tannath J. Scott, Steve Barrett, Ric Lovell

**Affiliations:** 1 School of Health Sciences, Western Sydney University, Sydney, NSW Australia; 2 Newcastle Falcons Rugby Club, Newcastle upon Tyne, UK; 3 Department of Sport and Exercise Sciences, Durham University, Durham, UK; 4 HIIT Science, Revelstoke, BC Canada; 5 French National Institute of Sport (INSEP), Laboratory of Sport, Expertise and Performance (EA 7370), Paris, France; 6 Kitman Labs, Performance Research Intelligence Initiative, Dublin, Ireland; 7 Institute for Health and Sport, Victoria University, Melbourne, VIC Australia; 8 Netball Australia, Melbourne, VIC Australia; 9 Carnegie Applied Rugby Research (CARR) Centre, Institute for Sport, Physical Activity and Leisure, Carnegie School of Sport, Leeds Beckett University, Leeds, UK; 10 Department of Sport Science Innovation, Playermaker, London, UK

## Abstract

Team-sports staff often administer non-exhaustive exercise assessments with a view to evaluating physiological state, to inform decision making on athlete management (e.g., future training or recovery). Submaximal fitness tests have become prominent in team-sports settings for observing responses to a standardized physical stimulus, likely because of their time-efficient nature, relative ease of administration, and physiological rationale. It is evident, however, that many variations of submaximal fitness test characteristics, response measures, and monitoring purposes exist. The aim of this scoping review is to provide a theoretical framework of submaximal fitness tests and a detailed summary of their use as proxy indicators of training effects in team sports. Using a review of the literature stemming from a systematic search strategy, we identified five distinct submaximal fitness test protocols characterized in their combinations of exercise regimen (continuous or intermittent) and the progression of exercise intensity (fixed, incremental, or variable). Heart rate-derived indices were the most studied outcome measures in submaximal fitness tests and included exercise (exercise heart rate) and recovery (heart rate recovery and vagal-related heart rate variability) responses. Despite the disparity between studies, these measures appear more relevant to detect positive chronic endurance-oriented training effects, whereas their role in detecting negative transient effects associated with variations in autonomic nervous system function is not yet clear. Subjective outcome measures such as ratings of perceived exertion were less common in team sports, but their potential utility when collected alongside objective measures (e.g., exercise heart rate) has been advocated. Mechanical outcome measures either included global positioning system-derived locomotor outputs such as distance covered, primarily during standardized training drills (e.g., small-sided games) to monitor exercise performance, or responses derived from inertial measurement units to make inferences about lower limb neuromuscular function. Whilst there is an emerging interest regarding the utility of these mechanical measures, their measurement properties and underpinning mechanisms are yet to be fully established. Here, we provide a deeper synthesis of the available literature, culminating with evidence-based practical recommendations and directions for future research.

**Supplementary Information:**

The online version contains supplementary material available at 10.1007/s40279-022-01712-0.

## Key Points

**Table:** 

Submaximal fitness tests provide a practical tool for team-sport practitioners to evaluate an athlete’s physiological state in a short duration and non-exhaustive manner, whereby testing can be integrated within the training session.
We propose a theoretical framework for submaximal fitness tests in team sports, encompassing an operational definition, five test protocol categories according to their exercise regimen and intensity parameters, and an evidence-based synthesis of protocols and outcome measures derived from three main response types: cardiorespiratory/metabolic, subjective, and mechanical.
Heart rate indices are most prevalent in the literature (57% of all outcome measures) and appear sensitive to detect positive endurance-oriented training effects. However, their utility in inferring negative transient effects associated with variations in autonomic nervous system function is questionable in team sports.
At present, limited evidence exists regarding the utility of ratings of perceived exertion to monitor training effects within submaximal fitness tests in team sports.
Collecting outcome measures derived from inertial measurement units during submaximal fitness tests may be a promising approach to monitor transient changes in lower limb neuromuscular function. Further work to establish the underlying theoretical framework and measurement properties is warranted.

## Background

Monitoring the training process and its outcomes within team-based sports requires a systematic approach that: (1) is grounded on a rigorous conceptual framework; (2) can be implemented pragmatically on a frequent basis; (3) uses proxy outcome measures possessing sufficient measurement properties; and (4) is sensitive to identify acute (e.g., post-match) and chronic (e.g., post-training program) training effects [1–4]. Such an approach can be used to inform decision making around athlete and training management, including future programming, adjustments to training delivery, or the implementation of recovery interventions [1].

Assessing aerobic-oriented training effects has traditionally been made via distinct maximal-effort exhaustive tests. For example, improvements denoted in maximal intermittent field tests (e.g., 30–15 Intermittent Fitness Test) can infer improved aerobic capacity (amongst other systems), whereas decreased values may be interpreted as a negative response or de-training [5, 6]. However, given the nature of the in-season phase common to professional teams, which frequently experiences fixture congested periods, it is considered less feasible to expose athletes to serial exhaustive assessments [7, 8]. With regard to neuromuscular function, a variety of test protocols are administered to quantify chronic training effects on athletic qualities such as strength and power, but more frequently as indicators of acute and transient responses (e.g., post-match recovery kinetics) [7, 9]. Similarly, the practicability of such assessments in the team-sports environment is challenged by different factors such as the number of athletes, the time available for discreet testing protocols, and the numerous contextual and individual elements that may undermine inferences derived from the data (e.g., motivation, physical qualities, season stage) [1, 7, 8].

Submaximal fitness tests (SMFT) have been proposed to deliver a feasible alternative to evaluate an athlete’s physiological state, presumably because of their time-efficient nature, low physical/physiological burden, and relative ease of administration [10]. In essence, SMFT provide a pragmatic and systematic approach of observing response(s) to a standardized physical stimulus [11, 12]. Such assessments have been investigated since the late 1940s [13] and were mainly adopted among clinicians diagnosing health conditions or physical fitness in non-athletic populations, whereby exposure to maximal or exhaustive activities was thought to be ill-advisable because of the health risk it posed to patients [13, 14]. Over the years, a number of walking [15–19], cycling [14, 20–22], and running [23–25] SMFT have been administered among clinical and healthy populations. These tests involve single or multiple continuous steady-state protocols, with some prescribing an absolute standardized intensity, while others include relative intensity ranges, or self-paced protocols (refer to File 1 in the Electronic Supplementary Material [ESM]).

### Elite Sports

The implementation of SMFT in elite sports has been traditionally used for quantifying relevant physiological transitions between exercise intensity domains (e.g., anaerobic threshold [i.e., the threshold indicates an equal rate of lactate production and disposal] and the onset of blood lactate accumulation [4-mmol·L^−1^ lactate threshold]) [26, 27], often administered to inform training prescription or determine exercise economy. However, these tests generally necessitate a laboratory environment, are resource intensive and obtrusive, and therefore considered less feasible in the day-to-day field context, particularly with large cohorts of athletes [7].

Individual endurance sport practitioners were the first to develop and implement SMFT as part of their training monitoring processes [28, 29]. Throughout the years, a broad range of cycling and running SMFT have been adopted across a variety of endurance sport athletes such as cyclists [28–32], runners [33–35], and triathletes [29, 36, 37]. Extensively used SMFT in endurance sports include exercise tasks prescribed by fixed internal intensities (% of an individual heart rate [HR] maximum) [32, 34], while the outcome measures are considered both external (e.g., speed [34], cadence, power [32] collected throughout the test) and internal responses (HR recovery [HRR] [38, 39], ratings of perceived exertion [RPE] [32] collected immediately post-exercise). Alternatively, researchers have adopted tests using standardized external intensities (usually via absolute running speed values) [33, 37]. Initially, the primary purpose of these SMFT was to predict performance (e.g., time trial) or physiological capacities (i.e., maximal oxygen uptake) [32]; however, more recently, they have been used to identify impaired performance (e.g., functional overreaching) [40, 41].

Because of the simplicity of implementing SMFT, their non-exhaustive nature, and their potential to provide information regarding both positive and negative training effects, SMFT have become common in team sports. Indeed, the adoption of SMFT in team-sports research [8, 10, 42] and practice [43, 44] has increased substantially over the last decade. However, given the broad range of SMFT adopted, including various protocols (continuous vs intermittent) [45, 46], activity modes (running vs cycling) [47, 48], intensities (fixed vs incremental, absolute vs individualized) [12, 48], outcome measures (cardiorespiratory/metabolic, subjective, or mechanical) [12, 49, 50], and purposes (monitoring positive vs negative effects) [47, 51], a synthesis appears warranted. Accordingly, the aims of this scoping review were to (1) develop an operational definition of SMFT and protocol taxonomy, (2) identify previously used SMFT in the team-sports research and discuss their conceptual and methodical aspects, (3) provide an audit of outcome measures, collection methods, and analytical processes, as well as evaluate the theoretical rationale underpinning their inclusion, (4) provide a narrative synthesis of the available research on SMFT as indicators of training effects in team sports, and (5) conclude with practical recommendations and future directions.

## Methods

Systematic searches of the electronic databases MEDLINE, Scopus, and Web of Science were used to identify relevant studies. From 2170 records identified in the original searches, we accepted 87 team-sport studies meeting our inclusion criteria. A detailed description of the searching strategy, screening process, and the inclusion–exclusion criteria are provided in File 2 in the ESM.

## Results

### Submaximal Fitness Test Definition

A table presenting the characteristics of the included studies is provided in File 3 in the ESM. Based on the available literature, we defined SMFT as a short exercise bout, undertaken at a standardized intensity that is intended to be non-exhausting, and performed with the purpose of inferring an athlete’s physiological state through the monitoring of relevant outcome measures. In this regard:

- *Exercise* is typically a cyclic activity involving large muscle groups. In team-sports settings, this is often administered as running activities, however, cycling has also been featured.
- *Standardized intensity* refers to a threshold(s) that is standardized based on an internal response or external intensity parameter, and can be either fixed for all athletes (i.e., absolute) or individualized to a capacity anchor (relative; e.g., fraction of HR maximum or maximal aerobic speed).
- *Non-exhausting* generally excludes frequent or prolonged ‘all-out’ maximal intensities, intensities that would cause voluntary cessation, or intensities that elicit an excessive training stimulus beyond that originally intended. From a practical standpoint, in team sports, the test should not have negative carry-over effects for the subsequent training session (for instance, if administered during the warm-up), or elsewhere it is implemented as an integrated standardized training component within the session plan (see for example, Sect. 3.2; intermittent-variable category).
- *Physiological state* can be defined as a particular condition or function of an individual’s physiological system, or a combination of systems—primarily, cardiovascular, respiratory, nervous, and muscular—at a specific point in time. In the context of SMFT, it may be used to infer an athlete’s current (physical) performance capacity or training effects (i.e., training responses).
  Training effects [4] indicate the direction (i.e., positive, negative) and the time course of the effect. Considering the challenges of dichotomizing time course criteria, we opted to use commonly referenced durations [4, 42] that align with the context and design of the included studies in our review, classified as acute (i.e., immediate [49] and up to a 1-week duration [47, 52]), short term (typically 1–3 weeks; e.g., congested or intensified periods [53, 54], training camps [54, 55], exposure to extreme environments [56, 57], season break [58]), and chronic (usually established over several weeks or months of training; e.g., pre-season [51, 59], training intervention [60, 61]). The first two are commonly referred as transient effects, while the latter typically indicates more ‘persistent’ or ‘enduring’ changes [4].
- *Outcome measures* include cardiorespiratory/metabolic, subjective, mechanical, or a combination, and are used as proxy (surrogate) measures that reflect (either directly or indirectly) the physiological systems they intend to assess. These are collected continuously within exercise and then aggregated into a summary metric (e.g., mean HR, accumulated ground impacts estimated via accelerometery-derived data), or measured immediately post-exercise (e.g., HRR, blood lactate, RPE).

### Protocol Taxonomy

**Fig. 1:**
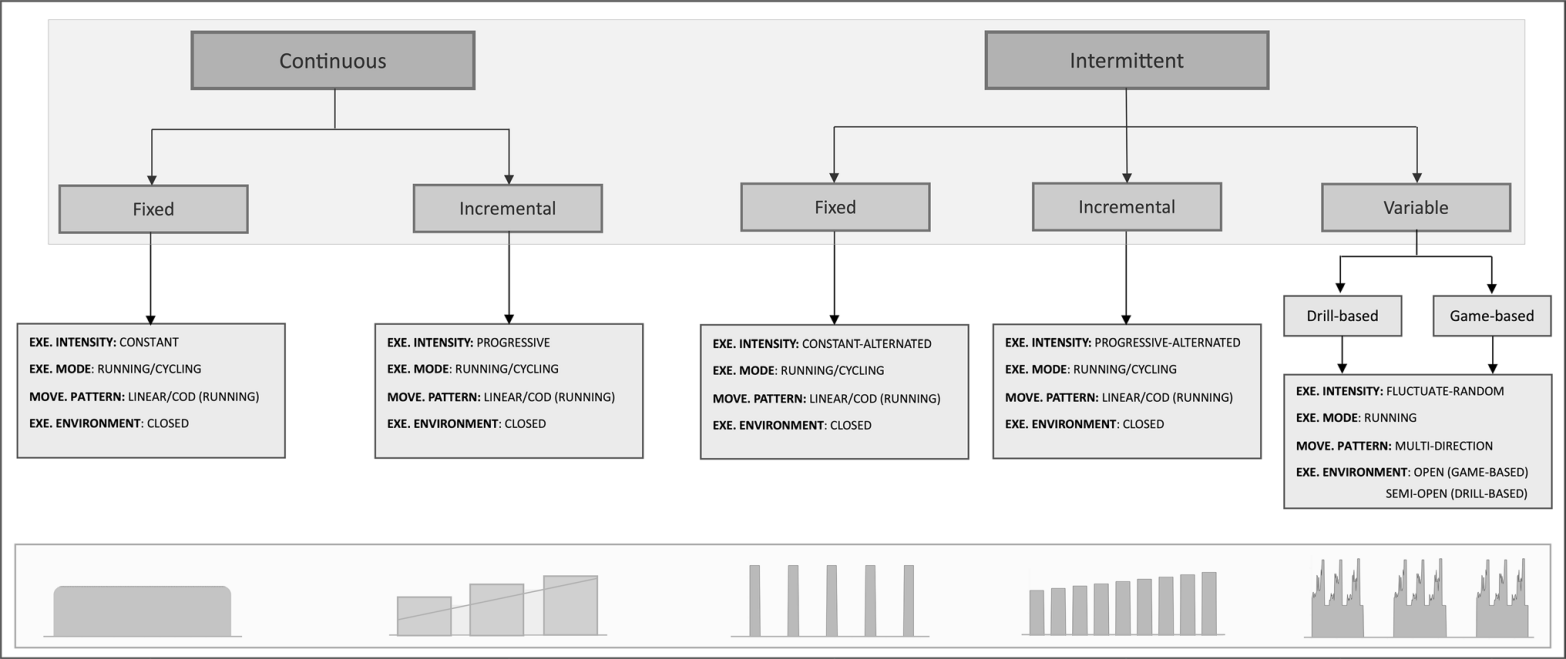
Submaximal fitness tests (SMFT) protocol taxonomy. Each protocol category consists of two levels: (1) exercise (EXE) intensity intermittency (continuous or intermittent) and (2) manipulation of exercise intensity (fixed, incremental, or variable), together yielding five distinct SMFT protocol categories (*shaded areas*). Intermittent-variable can be further categorized into drill-based and game-based formats. Each category can be further manipulated based on the movement (MOVE) pattern (linear, change of direction [CoD], and multi-direction), activity mode (running or cycling), and exercise environment (closed, semi-open, or open)

Information on SMFT protocols was extracted and categorized in reference to two main levels of classification: (1) exercise regimen (continuous or intermittent) and (2) manipulation of exercise intensity (fixed, incremental, or variable). Regarding exercise regimen characteristics, *continuous* activity represents a constant load exercise bout (typically for at least several minutes), without frequent alterations in velocity or rest periods [14, 62]. Alternatively, *intermittent* is defined as an activity that is interrupted and restarts after a particular time span, characterized by alternated loads and rest intervals [63, 64]. Considering all possible combinations of these categories, we subsequently identified five distinct SMFT categories from the available literature (Fig. 1), with each category further sub-divided based on the activity mode (running or cycling), movement pattern (linear, change of direction, or multi-directional), and exercise environment (closed, semi-open, or open):

- **Continuous-fixed** category represents a fixed-intensity exercise bout that remains constant for the entire SMFT and intends to elicit a steady state (e.g., 4 min running at 12 km·h^−1^) [65, 66].
- **Continuous-incremental** category is characterized by a progressive increase in intensity within (single) or between (multiple) exercise bout(s), whereas each bout lasts for several minutes (e.g., 4 min running with progressive increases in speed, 3 sets × 3-min bouts at 10, 11, and 12 km·h^−1^, interceded by 1-min rest periods) [11, 67].
- **Intermittent-fixed** category involves reoccurring activities performed at a constant intensity and rest intervals (e.g., four running bouts × 50–60 m at 18–22.5 km·h^−1^, separated by 30 s of recovery) [49, 68].
- **Intermittent-incremental** category predominantly involves fixed rest periods, while intensity is increased between exercise bouts (e.g., 30-s shuttle runs at 10–14 km·h^−1^, alternated by a 15-s rest period and with a-0.5 km·h^−1^ increment after each bout) [59, 69].
- **Intermittent-variable** category represents specific and non-specific standardized drills, and therefore locomotive demands fluctuate during the exercise (i.e., multi-directional movements). This category can be further categorized into drill-based and game-based exercises. Drill-based exercises refer to exercises that do not include competition features (e.g., passing drills) [70], whereas game-based exercises are characterized with competition features (small-sided games [SSG]) [71, 72].

#### Application of the Taxonomy to the SMFT Team-Sports Literature

**Fig. 2:**
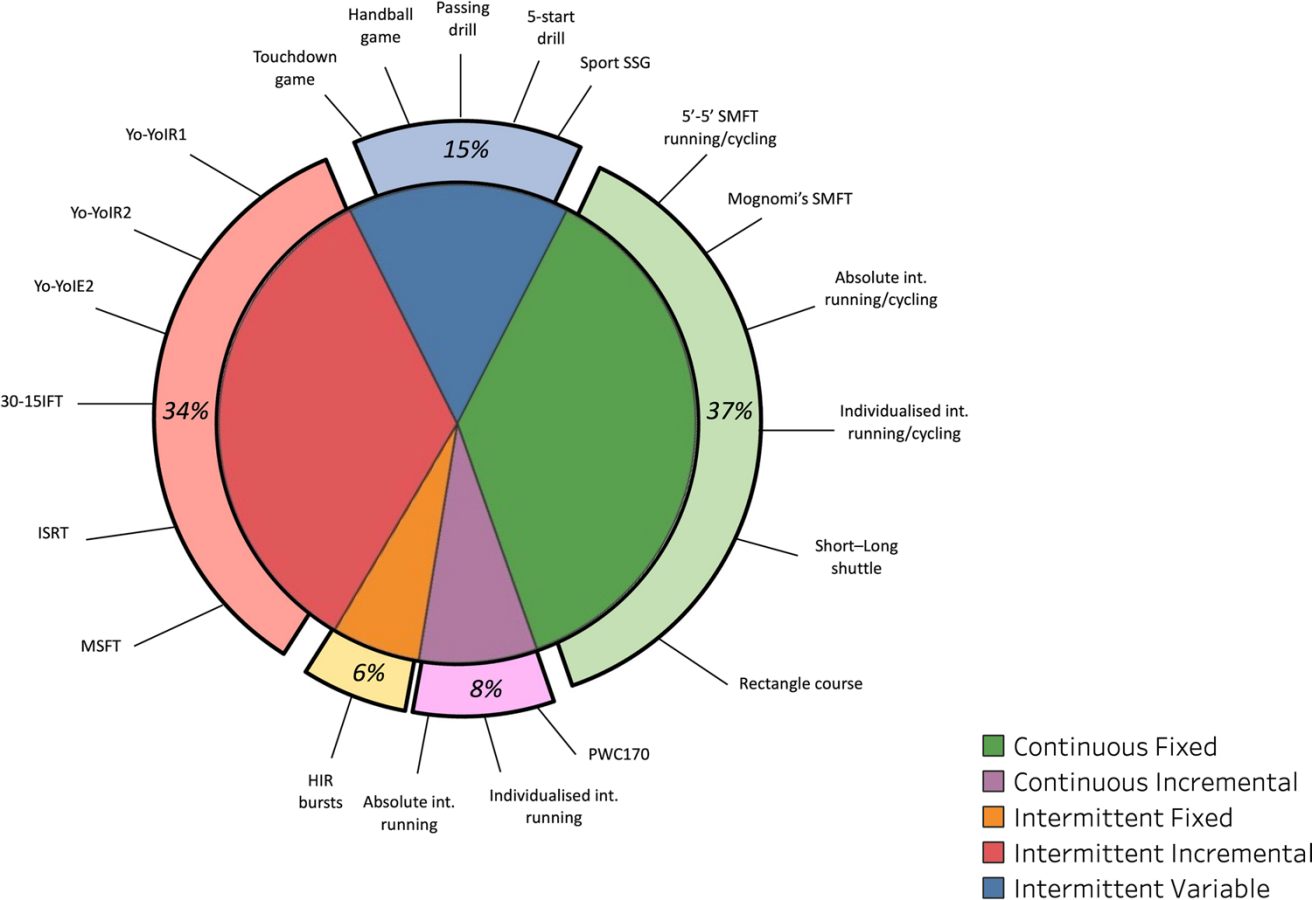
Frequency of submaximal fitness test (SMFT) categories and their highlighted individual tests as identified in the team-sports literature. A detailed description of these tests is highlighted in Table 1. *30-15IFT* 30–15 Intermittent Fitness Test, *HIR* high-intensity runs, *Int.* intensity, *ISRT* interval shuttle run test, *MSFT* multi-stage fitness test, *PCW* physical capacity work, *Yo-YoIE2* yo-yo intermittent endurance level 2, *Yo-YoIR1* yo-yo intermittent recovery test level 1, *Yo-YoIR2* yo-yo intermittent recovery test level 2

From the 87 included team-sport studies, we identified 100 independently described SMFT. As illustrated in **Fig. ****2**, the majority of studies in the literature adopted continuous-fixed SMFT (37%), followed by intermittent-incremental (34%), intermittent-variable (15%), continuous-incremental (8%), and intermittent-fixed (6%). Table 1 provides a summary of these SMFT as described in these studies. Continuous-fixed protocols were administered in both running and cycling exercise modes and include linear and change of direction (running protocols) movement patterns performed at absolute or relative standardized intensities. Continuous-incremental protocols comprised incremental exercises that were terminated when a specific internal (e.g., HR) or external (e.g., speed) intensity was achieved. Intermittent-fixed SMFT solely involved short-duration (8–12 s), high-intensity standardized bursts (~ 50–60 m). Intermittent-incremental SMFT incorporated shorter versions of the most common intermittent shuttle fitness tests, such as the Yo-Yo Intermittent Recovery Tests (Yo-YoIR1&2) [46] and 30–15 Intermittent Fitness Test [5]. Finally, intermittent-variable SMFT were mostly administered as game-based practices, including non-specific (handball, touchdown games) and specific (SSG of the sport) exercises, while drill-based practices included a variety of passing exercises (Table 1).

**Table 1:** SMFT described in the team-sports literature

SMFT				No. of studies	Test description				
Category	Activity mode	Move. Patt	Protocol		Dimensions	Stand. volume		Stand. intensity	Exe. configuration
Continuous- fixed	Running	Linear	5’-5’	1	Treadmill	5 minW/5 minR	833/917 m	10/11 km·h^−1^	
			Mognomi’s test	4	Track/treadmill	6 min	1350 m	13.5 km·h^−1^	
			5’-5’	2	Track	5 minW/5 minR	750 m	9 km·h^−1^	
			Individualized run	1	NS	6 min	1150–1200 m	60%V_IFT_	
			Individualized run	1	Treadmill	10 min	NS	90% V_AT_	
			Individualized run	1	NS	10 min	NS	75% HR_[RESERVE]_	
	Cycling		5’-5’	4	Bicycle	5 minW/5 minR		130 watts/85 rpm	
			Individualized cycling	1	Bicycle	4 min		75%VO_2_ max/70 rpm	
	Running	CoD	5’-5’ rectangle	2	20 × 20 m	5 minW/5 minR	750 m	9 km·h^−1^	≈37 turns 90°
			Rectangle course	3	100 × 50 m	4 min	800 m	12 km·h^−1^	≈10 turns 90°
			Short shuttle	5	20 m	3–5 min	750–1000 m	~ 9–12 km·h^−1^	≈38–50 shuttles 180°
			5’-5’	3	40 m	5 minW/5 minR	833–1083 m	~ 10–13 km·h^−1^	≈21–27 shuttles 180°
			Medium shuttle	2	40 m	5 min	833–1000 m	~ 10–12 km·h^−1^	≈21–25 shuttles 180°
			Long shuttle	3	80/100 m	5/4 min	1000/800 m	~ 12 km·h^−1^	12.5/8 shuttles 180°
			Long shuttle 5’-5’	2	*≈*80 m	5 minW/5 minR	750–1000 m	~ 9–12 km·h^−1^	≈9–12.5 shuttles 180°
			Individualized shuttle	1	57–68 m	4 min	≈680–820 m	75%MAS/ ~ 60%V_IFT_	12 shuttles 180°
Continuous- incremental	Running	Linear	Individualized run	1	Treadmill	NS	NS	S 8 km·h^−1^; F THR	↑ 1 km·h^−1^ every 1 min
			Absolute run	1	Treadmill	5 min	1000 m	S 8; F 12 km·h^−1^	↑ 0.8 km·h^−1^ every 1 min
			Absolute run	1	Treadmill	3 min × 3 sets	1700 m	9, 11, 14 km·h^−1^	3-min continuous bouts
			Absolute run	2	Treadmill	12 min	750–900 m	S 6/8; F 12 km·h^−1^	↑ 2 km·h^−1^ every 3 min
	Cycling		PCW170	1	Bicycle	3 min × 3 sets		F at 170 beats/min^−1^	120–170 beats/min^−1^ stages
	Running	CoD	Short shuttle	1	20 m	5 min	900 m	~ 8, 10, 12 km·h^−1^	1 min 8, 1 min 10, 3 min 12 km·h^−1^
			Short shuttle sets	1	20 m	4 min × 3 sets	2000 m	~ 9, 10, 11 km·h^−1^	≈100 shuttles 180°
			Medium shuttle sets	1	50 m	2 min × 4 sets	≈1600 m	~ 9.3, 11.1, 12.8, 14.6 km·h^−1^	≈32 shuttles 180°
Intermittent-fixed	Running	Linear	High-intensity run	3	50-m bursts	8 s	50 m	~ 18 km·h^−1^	3–4 reps
			High-intensity run	3	~ 60-m bursts	12 s	≈60 m	18–24 km·h^−1^	4 reps
Intermittent-incremental	Running	CoD	30-15_IFT_	1	40-m shuttle	≈8:25 min/ individualized	≈960 m/ individualized	F 13 km·h^−1^/ 25–75%V_IFT_	≈24/individualized shuttles 180°
			Yo-YoIR1	19	20-m shuttle	2–6:51 min	240–800 m	S ~ 10; F ~ 13.5/14.5 km·h^−1^	11–40 shuttles 180°
			Yo-YoIR2	3	20-m shuttle	2–8 min	240–1040 m	S ~ 13; F ~ 16.5–18 km·h^−1^	≈12–52 shuttles 180°
			*Mod.* Yo-YoIR2	1	18-m shuttle	2–4 min	≈470 m	S ~ 11.7; F ~ 14.9–15.8 km·h^−1^	
			Yo-YoIE2	2	20-m shuttle	2–6 min	280–920 m	S ~ 11.5; F ~ 13.5–14.5 km·h^−1^	14–46 shuttles 180°
			ISRT	1	20-m shuttle	2–9 min	≈260–1225 m	S ~ 10; F ~ 11–14 km·h^−1^	≈13–61 shuttles 180°
			Individualized ISRT	3	20-m shuttle	9–12 min	1225–1700 m	70% max of the test S ~ 10; F ~ 14–15 km·h^−1^	≈61–85 shuttles 180°
			MSFT	1	20-m shuttle	≈5:30 min	≈800 m	S ~ 8.5; F ~ 10.5 km·h^−1^	≈40 shuttles 180°
	Skating		30-15_IFT_ ice	1	40-m shuttle	≈3/6 min	≈390/870 m	S ~ 10.8; F ~ 12.7/15.2 km·h^−1^	≈10/22 shuttles 180°
			Yo-YoIR1 ice	2	20-m shuttle	6 min	≈700 m	S ~ 10; F ~ 14.5 km·h^−1^	≈35 shuttles 180°
Intermittent-variable GB	Running	MD	9v8 handball	1	141 m^2^/player	8 min			Similar game style
			4v4 touchdown	1	150 m^2^/player	4 min of 1 set			Score into the zone area
			4v4 SSG	1	80 m^2^/player	4 min × 3 sets			
			4v4 SSG	1	120 m^2^/player	1.5 min × 6 sets			With GK
			4v4 SSG	1		2 min × 4 sets			
			5v5–10v10 SSG	1	117 ± 65 m^2^/player	NS			Possession/with GK
			3v3–6v6 SSG	3	67.5–90 m^2^/player	3 min × 2 sets			With/without GK, limit/free touch
			5v5 + 5 SSG	1	135 m^2^/player	3 min × 4 sets			With GK, free play
			5v5 SSG	2	62.5/105 m^2^/player	3 min × 3–4 sets			Without GK, free play
Intermittent-variable DB	Running	MD	5-star drills	2	15–25 m runs	4–5 min × 3 sets			Kicking, handballing, and marking
			Passing drill	1	~ 2/3 pitch	6 min × 2 sets			Short and long distances

*30-15*_*IFT*_ 30–15 Intermittent Fitness Test, *CoD* change of direction, *DB* drill-based, *F* finish, *GB* game-based, *GK* goalkeeper, *HR*_*[RESERVE]*_ HR reserve, *ind.* individualized, *ISRT* interval shuttle run test, *m* meters, *MAS* maximal aerobic speed, *max* maximum, *MD multi-direction*, *min* minute, *Mod.* modified, *Mov. Patt.* movement pattern, *MSFT* multi-stage fitness test, *NS* not specified, *PCW170* physical work test, *R* recovery, *reps* repetitions, *rpm* rate per minute, *S* start, *sec* second, *SMFT* submaximal fitness tests, *SSG* small-sided games, *THR* target HR = [(HR maximum – resting HR) * 80–85% intensity] + resting HR, *V*_*AT*_ velocity at anaerobic threshold, *V*_*IFT*_ final velocity achieved in 30-15_IFT_, *W* work, *Yo-YoIE2* Yo-Yo intermittent endurance level 2, *Yo-YoIR1* Yo-Yo intermittent recovery test level 1, *Yo-YoIR2* Yo-Yo intermittent recovery test level 2, *≈* approximately, ~ mean, *↑* increase, ° CoD angle

SSG area per player was computed including GK

## Outcome Measures

**Fig. 3:**
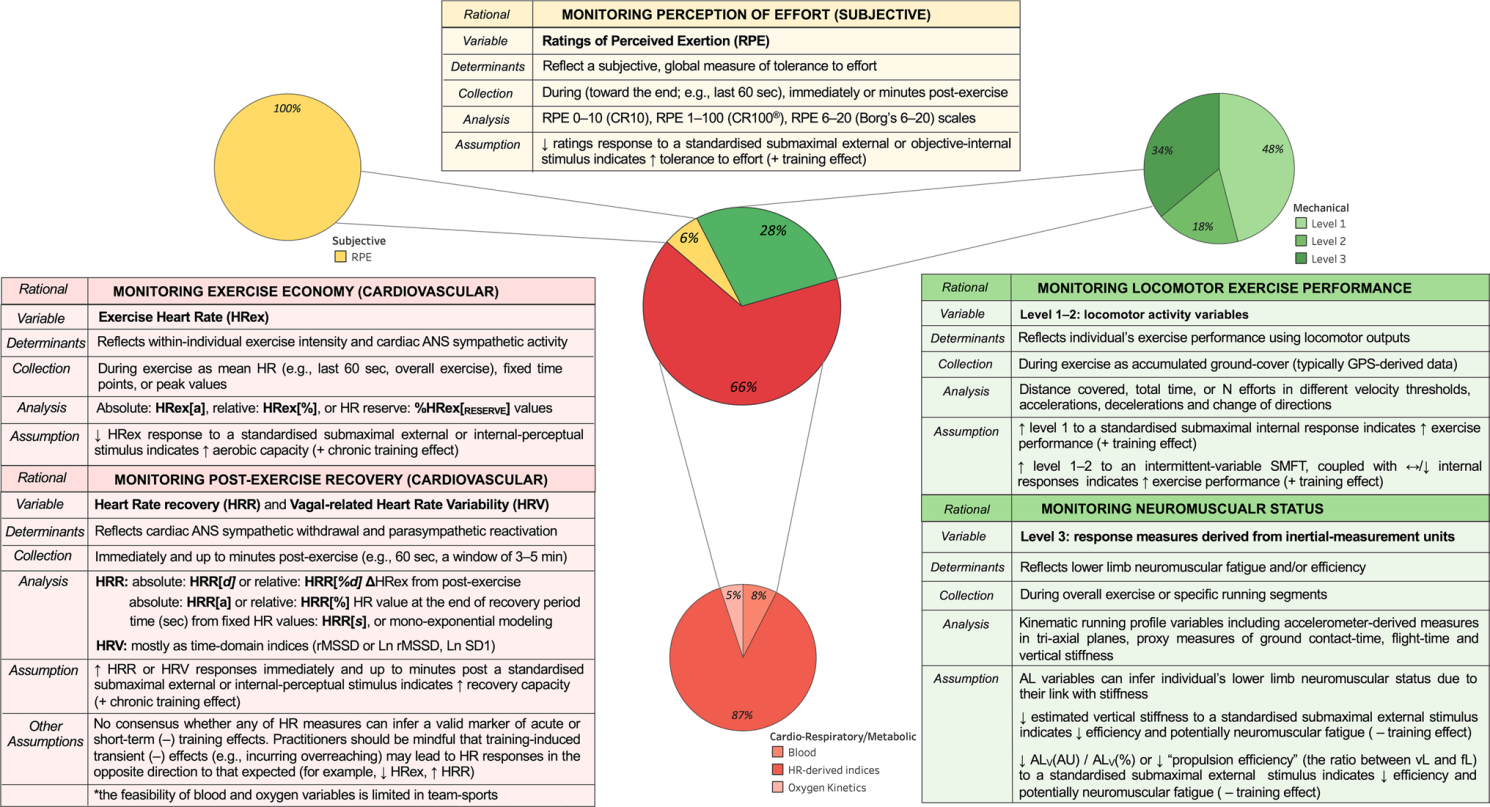
Frequency of submaximal fitness test (SMFT) outcome measures as identified in the team-sports literature. Heart rate (HR)-derived indices are the most common cardiorespiratory/metabolic outcome measures and include variables representing exercise intensity (HRex) and recovery (HRR and HRV). Level 1–2 mechanical outcome measures represent locomotor activity variables collected during SMFT that are standardized by an internal stimulus or intermittent-variable exercise such as small-sided games (SSG). Level 3 mechanical outcome measures are response measures derived from inertial measurement units (micro-electrical mechanical systems [MEMS]) for monitoring neuromuscular status. Subjective outcome measures represent tolerance to effort and have been monitored solely via ratings of perceived exertion (RPE). *AL* accelerometry-load, *AL*_*V*_* (AU)* the vertical vector magnitude component of tri-axial *AL, AL*_*V*_* (%)* percentage contribution of the vertical vector magnitude component to tri-axial *AL, ANS* autonomic nervous system, *CT* contact time, *Force-load (fL)* sum of estimated ground-reaction forces during all foot impacts, *GPS* global positioning system, *Ln rMSSD* natural log of rMSSD, *Ln SD1* log-transformed standard deviation of successive R spikes measured from Poincaré plots, *rMSSD* root mean square of the sum of all differences between successive normal heartbeats, *Velocity-load (vL)* sum of distance covered weighted by the speed of displacement (in SMFT refers to the actual mean velocity), + positive, − negative

We identified 202 total outcome measures used in previous team-sports research. As shown in Fig. 3, cardiorespiratory/metabolic were the most used outcome measures (66%), followed by mechanical (28%) and subjective (6%). The following sections present the outcome measures corresponding to each response type, discuss their putative underlying mechanisms, and synthesize the current available evidence examining their changes within the SMFT framework.

### Cardiorespiratory/Metabolic Outcome Measures

In team-sports settings, which can include a large number of individuals who may possess different aerobic capacities [8, 12], it is difficult to implement SMFT that are standardized by internal intensity variables. Accordingly, the majority of the tests were applied by standardizing the external intensity and measuring the corresponding internal responses [10, 73]. A variety of cardiorespiratory/metabolic outcome measures have been used in the literature, with the most common being HR-derived indices (Fig. 3). These include variables collected during (exercise HR [HRex]), immediately after (HRR) the SMFT, and soon after (HR variability [HRV, vagal-related HRV]) the SMFT. Other measures include blood markers (e.g., blood lactate) [67, 74–80] and oxygen consumption-related parameters (e.g., oxygen uptake) [75, 76, 81, 82]. As blood and oxygen uptake outcome measures are time consuming, expensive, and obtrusive, their viability to provide standardized and repeatable response measures is considered limited, particularly in team sports [11, 83]. Accordingly, we focused on HR-derived indices.

#### Exercise HR

Exercise HR is collected during the SMFT and is analyzed in absolute (beats per minute; HRex[a]) [47, 59, 66, 69, 74–76, 81, 82, 84–103, 104], relative to maximal HR (HRex[%]) [6, 11, 12, 45, 46, 48, 51, 54, 56, 57, 59–61, 66, 67, 70, 79, 83, 84, 89, 91, 105–119, 120], or HR reserve (HRex[reserve]) [53] values. A variety of methods are used to derive HRex, with the majority calculating the mean HR during the last 10–60 s of the test [11, 12, 45, 47, 48, 51, 53–61, 66, 69, 71, 74, 75, 82, 83, 85–87, 89, 92, 93, 96, 97, 103, 105–108, 111, 113–115, 117, 119–121]. Other approaches calculate the mean HR during the overall test (particularly during intermittent-variable protocols such as SSG) [70, 71, 79, 94, 98, 101, 104, 116], specific fixed timepoints [6, 46, 59, 60, 84, 91, 95, 99, 105, 109, 110, 112, 122], or peak values observed [61, 90].

#### HR Recovery

Heart rate recovery can be defined as the rate at which HR declines after exercise cessation [10, 39] and may be collected with athletes lying supine [123], sitting upright [12, 45], standing [89, 121], or walking [95]. Similarly, HRR can be assessed as the absolute (HRR[*d*]) [45, 47, 48, 53, 54, 57, 59, 61, 66, 88, 100, 103, 107, 114, 119, 121, 123, 124] or relative (HRR[%*d*]) [12, 47, 53, 60, 66, 102, 104, 107, 113, 114, 121] difference between HRex and HR value observed between 10 and 180 s post-test. Alternative approaches include the actual HR value observed at the end of the designated recovery period in absolute (HRR[a]) [66, 74, 89, 95, 99, 121, 125] or relative (HRR[%]) [66, 89, 109, 110, 113] values, or the overall mean HR during a variety of fixed time intervals [90, 97, 107]. Other approaches calculate time-based variables such as the time required to decrease from between fixed HR values (HRR[*s*]; e.g., time between 80 and 70% HR maximum) [79], or the time constant of HRR derived from mono-exponential modeling [61, 126].

#### HR Variability

Vagal-related HRV is defined as the variability in the time intervals (usually in milliseconds) between adjacent heartbeats and reflects the regulation of cardiac autonomic nervous system (ANS) balance [10, 127]. Heart rate variability indices are commonly collected in a seated or supine position and resting state in a laboratory or quiet room [10, 42]; however, as the aim in SMFT is to monitor the response during and after a given submaximal workload, only HRV parameters observed immediately or soon after the test are relevant for SMFT in team sports. These measures are usually analyzed within a window of 3–5 min post-test cessation, and predominantly calculated as time domain-related variables such as the square root of the mean of the sum of the squares of all differences between successive normal heartbeats (rMSSD) [61, 107] or its natural log (Ln rMSSD) [45, 47, 53, 59, 100, 106, 114], natural log of standard deviation of successive ‘R spikes’ (the peak of the QRS complex, reflective of ventricular depolarization, recorded from an electrocardiogram wave) measured from Poincaré plots (Ln SD1) [57, 106], and the standard deviation of mean interval differences between normal heartbeats (SDNN) [61, 107].

#### Putative Mechanisms

Heart rate-derived indices are commonly used to inform chronic aerobic-oriented training effects, attributed to the linear relationship between HRex and oxygen uptake during an intended steady-state activity [10, 42]. A reduction in HRex to a standardized submaximal stimulus may represent improved exercise economy, which may translate to the development of aerobic fitness [51, 120], whereas an increment of HRex is considered to reflect a negative response (de-training of the cardiorespiratory system) [87], likely due to central adaptations (i.e., left-ventricular function) [128]. In addition, an increment of HRR or vagal-related HRV measures is considered a positive effect [59, 60, 107], reflecting the reactivation of the parasympathetic system and hemodynamic adjustments post-exercise [10]. That said, in addition to being more time consuming, these measures may be influenced by preceding exercise, with higher intensities eliciting increased blood acidosis that simulate the metaboreflex, and therefore may reduce HR decay post-exercise and alter HRR and vagal-related HRV results [10, 53].

The use of HR-derived indices to infer negative transient training effects is inconclusive in the SMFT research. The theoretical basis for their inclusion is due to the potential influence of various training-induced physiological processes that originate in central (i.e., ‘central command’) and peripheral (e.g., afferent feedback from skeletal muscles) body regions to alter cardiac ANS function (i.e., the balance between the sympathetic and parasympathetic systems), and subsequently HR activity [10, 38, 42]. It has been hypothesized that training-induced fatigue or an incomplete recovery might result in a greater muscle activation at a given intensity [129], promoting increased oxygen demands [129] and yielding accelerated cardiac sympathetic activity that consequently increase HRex, and reduce HRR and HRV [114, 129]. In contrast, previous research has proposed that increased training stress (leading at least to an overreaching state) may cause opposite responses — increased parasympathetic activity or blood plasma volume, consequently lowering HRex and increasing HRR and HRV [37, 38, 130].

#### Inferring Physiological State

Generally, across a standard micro-cycle, HR measures tend to stay relatively stable [45, 47]. For example, studies among youth and senior athletes have observed no changes in the day-to-day variability of SMFT HRex [45, 47, 75] and HRR [45, 47] derived from multiple SMFT administered throughout the week, despite substantial variations in training loads. Whilst vagal-related HRV indices are considered to provide a better insight into the cardiac ANS [10, 53], daily variations in training loads were associated with stable [45, 47] and lower [53] responses. The disparity across studies may be due to the differences in training loads and HRV variables [53], an athlete’s fitness levels [45], or simply reflective of measurement ‘noise’ (either measurement errors or biological variations) [10, 61, 107], thus challenging between-study comparisons.

The current research suggests that the use of HR-derived indices appears more relevant after acute (3–7 days) and short-term (~ 2 weeks) altered training stress, albeit it may cause misleading interpretations. In team sports, 3–4 consecutive days of accumulated training loads have been associated with both increased [114] and decreased [54] SMFT HRex. Lower SMFT HRex values have been also observed after 2 weeks of intensified training [84], but also following a substantial decline in training loads due to season breaks [58]. Whilst the information in team-sports settings is limited, studies among various cohorts of individual endurance athletes (e.g., cyclists, triathletes) provide encouraging evidence that short-term intensified periods (1–3 weeks) of training-induced fatigue (incurring functional overreaching) lowered SMFT HRex [36, 38, 130] and increased SMFT HRR [36, 37, 39]. These responses likely reflect a complex interplay between acute cardiac ANS function (usually referred to as larger parasympathetic activity) and increased plasma volume [37, 54, 104], leading to an enhanced stroke volume for a similar cardiac output [10, 131]. In summary, the use of HR-derived indices (especially HRex and HRR) to infer transient training effects associated with cardiac ANS dysfunction is currently questionable (at least, not straightforward), with no consensus around the underlying mechanisms and conflicting results in the literature.

Overall, SMFT HRex has small to very large inverse relationships with performance indicators (i.e., maximal oxygen uptake, intermittent endurance capacity) when measured concurrently (lower HRex is associated with higher test results) [46, 48, 105], suggesting its validity as an indicator of an individual’s current endurance capacity. Indeed, it has been highlighted that a chronic exposure to internal (e.g., HR-based training impulse, session RPE) [86, 90] and external (e.g., total distance covered, force load) [71, 120] loads is associated with reduced SMFT HRex. However, the studies examining training effects within athletes have reported contrasting findings. For example, studies in soccer players [46, 91] have observed moderate to very large relationships between SMFT HRex and intermittent endurance performance at different timepoints across the season, albeit with no interaction between the magnitude of these effects. Likewise, improved [91] or maintained [87] intermittent running ability did not necessarily coincide with reduced or stable SMFT HRex, respectively. In contrast, studies conducted on a variety of team sports and age groups have observed significant relationships between changes in similar markers from pre-season to in-season [51, 59], and across a full season [107]. Improved SMFT HRex were also largely correlated with the changes in running speed at 4 mmol·L^−1^ blood lactate [11, 83] or a reduced oxygen uptake at fixed submaximal intensities (i.e., exercise economy) [81].

Research observations are more consistent where SMFT HRex has been administered to evaluate the adaptation time course to changes in extreme environments (heat and altitude) during training camps or competitions [55–57, 93, 104, 106, 132]. Collectively, HRex displayed substantial deteriorations upon arrival and up to days of exposure [56, 57, 93, 104], and generally return to baseline values within 6–10 [55, 106] or 14 days [56], with quicker adaptations among highly trained individuals or across repeated exposures within individuals [133, 134]. Taken together, whilst it appears that SMFT HRex has the potential to serve as a valid and sensitive marker of positive training-induced effects, it remains questionable whether it can be used as a surrogate measure of within-athlete changes in maximal aerobic capacity. There is, however, considerable evidence suggesting its use during exposure to changes in environments for monitoring the athlete’s acclimatization.

The magnitude of correlations between cardiac parasympathetic-related variables (HRR and vagal-related HRV) and performance indicators is less clear and ranged from no correlation to a very large relationship [48, 66, 100]. The disparity could be the consequence of varied protocol intensities [10, 35, 42, 45], collection time (e.g., 60 s and 180 s post-exercise) [48], analysis approaches [66], and fitness criterion measures [45, 79]. Importantly, inferences regarding their long-term validity as proxy measures of chronic training effects can be somewhat impacted by SMFT intensity (i.e., HRex) [10]. The rate of the sympathetic withdrawal and parasympathetic reactivation post-exercise is altered when the recovery period starts from different intensities. As an example, different absolute HRR values (HRR[*d*]) may be expected 60 s post-SMFT if the exercise intensity varies (e.g., 90% vs 75% HR maximum). Hence, in theory, a significant reduction in HRex might influence the concurrent interpretation of post-exercise outcomes. Although this issue has been addressed by analyzing HRR in relative values (HRR[%*d*]) [60, 114], the time necessary to decrease from two fixed HR values (HRR[*s*]) [79], or employing individualized intensity protocols [12], these are not yet fully understood. In support of this, regardless of the HRR analysis used, authors examining training-induced changes in both HRex and HRR did not always find congruent trends [60, 61, 102]. Other studies observed a lack of association between changes in HRR and endurance performance, despite significant relationships with HRex [59, 107]. However, increased SMFT vagal-related HRV has been shown to be more appropriate, with studies reporting its validity for monitoring endurance-oriented training effects [59, 61, 107].

#### Considerations

Of critical importance when using HR-derived indices as proxies of training effects is the range of confounding factors, such as environmental (e.g., temperature), habitual (e.g., sleep, diet), circadian (time during the day), and psychological (e.g., emotions, stress). These could all contribute to the error of measurement of SMFT HR measures — HRex (coefficient of variation (CV): 1.0–3.5%) [12, 45, 48], HRR (CV: 2.8–13.8%) [45, 48, 66], and vagal-related HRV (CV: 6.6–19.0%) [45, 61, 106] — and should be considered (or standardized where pragmatic) when interpreting changes in HR-derived indices responses to SMFT [10, 42].

Further research is warranted to explore the use of all HR-derived measures to infer acute and short-term effects, in particular, verifying the interaction between temporary changes in cardiac ANS function and plasma volume responses. In this respect, it should be highlighted that HR responses may still be less appropriate to denote peripheral neuromuscular fatigue, which are considered more important to monitor delayed recovery and injury risk mitigation in team sports [49, 135]. In the longer term, HRex is probably the easiest to collect and most reliable HR measure, and its utility in observing positive changes in aerobic capacity has stronger empirical support. Accordingly, the utility of adding post-exercise (HRR, vagal-related HRV) SMFT HR responses to infer chronic effects may be redundant. In order to enhance interpretations, future research should first determine meaningful changes in SMFT HRex (i.e., smallest worthwhile change) in reference to variations in physiological states.

Finally, when monitoring the responses to intermittent-variable SMFT (e.g., SSG), an athlete’s HRex may be influenced by their locomotor activity. These are likely to differ between tests and should be accounted for when interpreting data. Therefore, we recommend quantifying intermittent-variable SMFT locomotor activity for consideration. Given the reasonable association between internal and external measures during field-based sessions [136], it is also possible to standardize HRex to a given (fixed) external intensity parameter. Whilst some have attempted to achieve this by dividing the former by the latter, creating a ratio [137], there are complex statistical properties and assumptions associated with such indexes, presenting as a major validity concern [138]. To appropriately examine HRex while holding external intensity parameters constant, we recommend linear regression techniques, which do not violate statistical assumptions and achieve the desired outcome ratios [139].

### Subjective Outcome Measures

Subjective measures are recognized by their ability to serve as gestalt measures that can be used across different exercise typologies, given their feasibility and low cost [140]. These are commonly applied to quantify an athlete’s perception of intensity and training effects. The former is derived solely from RPE, while the latter are commonly referred to as athlete-reported outcome measures [141] of latent response constructs such as readiness, wellness, and stress. [4, 142]. Accordingly, RPE are the only subjective outcome measures that can be applied to SMFT, a notion supported by their exclusivity (albeit the limited number of studies) in the team-sport SMFT literature (Fig. 3). Among the available studies, different scales such as the Category-Ratio 10 (CR10 deciMax) [50, 56, 57, 67, 70, 98, 108], 100 (CR100® centiMax) [91], and 6–20 (Borg’s 6–20) [75, 76] have been adopted, using a variety of collection protocols (during the last 180 s, immediately, and up to 5 minutes post-exercise).

#### Putative Mechanisms

Perception of effort is defined as the ‘conscious sensation of how hard or strenuous a physical task is’ [142], and mainly depends on how easy or hard it is to breathe and drive the working muscles during exercise [143]. As part of SMFT, the athlete provides a retrospective appraisal of perceived effort to a standardized stimulus that can be prescribed by either an objective internal [32] or external [91] means. Because RPE is strongly associated with cardiorespiratory, metabolic, and neuromuscular measures of exercise intensity [144, 145], and influenced by the mental state [142], changes in RPE may reflect positive [32, 91] or negative [37, 41] alterations in the psycho-physiological state. However, given their gestalt nature, it is perhaps difficult to align RPE as a proxy to a single physiological system during SMFT. For example, RPE has been empirically associated with HRex during a continuous exercise [144] and might therefore be used as a cardiorespiratory proxy measure. However, spinal or supraspinal motoneuron inhibition, which is a neuromuscular phenomenon, can increase central motor command and subsequently RPE [146]. This is not to say that RPE cannot be used to infer a physiological state during SMFT, but rather the mechanisms may be less precise. In addition, given that RPE has also been used to regulate exercise intensity [147], RPE can be used as an anchoring intensity variable (i.e., running or cycling at fixed RPE), whereby external outcomes such as velocity or power output are used as response measures [148]. However, to our knowledge, there is no published team-sports research evaluating the theoretical basis of such SMFT and their actual utility.

#### Inferring Physiological State

Whilst some evidence supports the use of SMFT RPE [58, 91, 98], it is difficult to support or refute their utility to determine training effects, given the limited data available in team sports. In endurance athletes, RPE have been shown to detect negative transient effects associated with functional overreaching and disturbances in endurance performance [30, 37, 41], while their sensitivity to chronic positive effects is less certain [34, 40]. In the team-sports context, one study [91] showed that reduced SMFT RPE (albeit maintained HRex[a]) was accompanied with enhanced intermittent running performance (Yo-YoIR1) and soccer match high-intensity running [91]. Another study [50] in professional soccer players did not observe any significant relationships between RPE collected immediately after an individualized SMFT and athlete-reported outcome measures across 6 in-season standard weeks. Despite the conflicting results, researchers have suggested the potential usefulness of SMFT RPE when measured concomitantly with other objective outcome measures (e.g., HRex) as part of a multivariate monitoring approach [37, 42, 130].

#### Considerations

It is noteworthy to highlight some of the challenges associated with collecting and interpreting RPE in team sports, where interpretation is challenged by the presence of the coach and peers biasing ratings, the application of unvalidated collection tools, lack of or inappropriate athlete familiarization/education, and their gestalt nature [142, 146, 149]. Moreover, RPE may be confounded by other sensations associated with exercise, such as mood, discomfort, pain, and enjoyment [142]. In view of these challenges, researchers may consider other perceptual measures such as ratings of fatigue [150], or techniques such as numerically blinded [149] and differential [151] RPE, alongside the implementation of rigorous familiarization processes to facilitate authentic and sensitive perceptual ratings associated with SMFT.

With this in mind, a benefit of SMFT in team sports is to provide an assessment that can be seamlessly integrated into the training session such as the warm-up or standardized drills. The need to collect RPE from all athletes individually (~ 20–40) under controlled conditions is likely to be disruptive and impractical, which is perhaps why there are few studies using this practice. It is probably reasonable to assume that unless SMFT are completed as a discreet activity, with smaller groups, or RPE collection procedures are made more accessible (e.g., mobile devices, human resources), these outcome measures may be less pragmatic or sustainable in team sports.

### Mechanical Outcome Measures

Within the current monitoring schemes in sport, there is a broad classification of external load parameters. These indices represent kinematic outputs [152] performed by an athlete throughout an exercise bout/session [153] and have been classified into three distinct levels, which we will use here to audit their implementation in the context of SMFT [8, 154].

#### Level 1–2 Measures

*Level 1* variables are typically the locomotor performance outputs including distance covered, time spent, or the count of efforts in different velocity zones, whilst *Level 2* variables reflect changes in velocity such as accelerations and decelerations (i.e., change of directions) [8]. Such kinematic parameters are routinely collected using global positioning systems (GPS) or other tracking technologies (i.e., semi-automated pixel tracking, local positioning systems). In the team-sports SMFT framework, the use of level 1–2 mechanical outcome measures can occur in two scenarios: (1) monitoring the speed achieved to a submaximal exercise that is standardized by internal intensity responses [50, 155] and (2) monitoring the changes in these variables during intermittent-variable standardized drills [54, 58, 70–72, 79, 93, 98, 106, 116, 132, 156], as they are standardized by a variety of other parameters such as duration, sets, recovery, and unique constraints (e.g., number of players, rules modifications). Whilst the former is considered less practical in team sports for many pragmatic reasons, the latter are implemented as a part of the training plan, encompassing sport-specific actions and are perhaps the most feasible to apply routinely [8, 131]. Conceptually, higher values (e.g., accumulated distance covered), coupled with stable or lowered internal responses are indicative of positive effects (i.e., improved exercise performance) [8, 58, 98]. In fact, studies examining level 1–2 variables during drill (passing) and game (SSG) exercises have highlighted their pragmatic advantages to deliver information related to an athlete’s performance [58, 71, 72, 98]. However, it should be noted that intermittent-variable SMFT are influenced by a variety of individual and contextual factors such as technical level, motivation, and tactics [8, 72], and have a higher degree of variation (test–retest reliability) compared with other SMFT modalities [70, 72, 156, 157], and therefore, should not necessarily be interpreted in the aforementioned simplistic manner [8].

#### Level 3 Measures

*Level 3* external load variables are derived from inertial-measurement units such as tri-axial accelerometers, magnetometers, and gyroscopes [8] (collectively referred to as micro-electrical mechanical systems [MEMS] [158, 159]). Unlike level 1–2 variables, these outcome measures can be used for the majority of SMFT applied in the team-sport context and have been proposed to provide an insight into an athlete’s neuromuscular system, given their potential link with lower limb vertical stiffness [135, 154, 160–163]. Vertical stiffness is considered to affect several athletic parameters, including elastic energy storage and utilization (i.e., stretch shortening cycle) [164] and has traditionally been measured through a variety of jump assessments (counter-movement, hopping, and drop jumps) using variables such as jump height, contact time, and flight time [52, 165, 166]. However, because of the limited viability of these assessments in field conditions and their lack of specificity (jumping activities may be less sensitive to detect changes in running strategies) [52, 161], researchers and practitioners have started to collect proxy variables required to estimate vertical stiffness derived from MEMS during SMFT [49, 52, 55, 65, 68, 72, 167–169].

To date, studies have adopted accelerometer-derived vector magnitudes (collectively termed in this review as accelerometery load [AL]) [49, 52, 72] and individual vector components (vertical AL [AL_V_], antero-posterior AL [AL_AP_], and medio-lateral AL [AL_ML_]) using MEMS-embedded accelerometers [49, 52, 68, 167, 168], predominantly collected during intermittent-fixed protocols comprising high-intensity running bursts [49, 52, 55, 68, 168]. Generally, reduced AL in the vertical plane (AL_V_ [arbitrary units; AU]), or the percentage contribution of the AL_V_ to the overall tri-axial vector magnitude (AL_V_ [%]) during SMFT have been postulated as an indicator of reduced leg vertical stiffness and subsequently inferred a degree of lower limb neuromuscular fatigue [167, 170]. Theoretically, reduced vertical stiffness may lead to reduced efficiency for the same speed through altered kinetic and kinematic parameters, such as reduced vertical ground-reaction forces and increased ground-contact time [161, 170], which likely lead to decreased stride length [52, 72] and elevated energy cost [162, 170].

#### Inferring Physiological State

Studies investigating acute effects on mechanical outcome measures during SMFT are scarce, and those available are quite disparate in terms of protocols, variables, and their analytical processes [49, 52, 65]. Nonetheless, there is an emerging agreement from these studies that suggest AL measures can provide sensitive indicators of an athlete’s neuromuscular fatigue and efficiency. In a group of professional soccer players who performed an intermittent-fixed SMFT (4 ×  ~ 60-m runs, alternated by an ~ 30 s recovery) before and immediately after a training session, Buchheit et al. [49] found that various AL variables respond differently to different training modes (strength, speed, endurance-oriented conditioned sessions). Estimated vertical stiffness slightly increased after all training modes, whereas propulsion efficiency (the ratio between velocity loads and force load; refer to Fig. 3 for variables) was session dependent (largely increased after strength, small and moderate decreases after endurance and speed, respectively), suggesting its sensitivity to detect changes in running strategies (hypothetically, horizontal force application capability) [8, 49]. A study [52] using a similar SMFT protocol and outcome measures among university rugby union players reported large relationships between the vector magnitude and vertical accelerometer components derived from the constant phase of the run, versus leg stiffness measured more directly via submaximal hopping test performed on a force platform [52]. Whilst only trivial effects were observed in leg stiffness over the week, the changes in SMFT AL data were large [52].

Similar trends were also found in a study investigating the alterations in triaxial AL data collected before, 48 h, and 96 h after an Australian football match [68]. A main finding reported in this latter study was that AL_V_ (AU) and AL_ML_ (AU) derived from the constant phase of the SMFT were still impaired 96 h post-match among players who were classified as ‘fatigued’ (> 8% reduced counter-movement jump at a 48 h post-match) [68]. Finally, a within-individual longitudinal study among soccer players [72] showed reductions in AL m·min^−1^ and AL slow·min^−1^ collected during a standardized SSG (5v5 + 5) 1 day before a match were concomitant with a reduction in neuromuscular function (flight-time:contact-time ratio measured from counter-movement jump) and an altered match running profile—increased AL_ML_ (%) and decreased AL_V_ (%) contribution to AL—indicative of potential neuromuscular fatigue [72, 167]. Collectively, it appears that specific mechanical level 3 metrics may be useful for identifying acute variations in performance (neuromuscular fatigue and efficiency) associated with changes in lower limb function.

Inferring longer term training effects from mechanical variables ascertained during SMFT has received limited attention, and insights have been drawn exclusively from tracking locomotor outputs during intermittent-variable SMFT in the form of SSG. For example, moderate to very large positive relationships have been reported between higher level 1–2 outputs during SSG and intermittent running capacity [98]. In addition, during intensified training camps (1–2 weeks), within-individual increases in running parameters (e.g., total and high-speed running distance) measured during intermittent-variable SMFT were also concordant with improved intermittent running capacity [54, 106, 132]. Of note, the utility of all mechanical outcome measures (levels 1, 2, and 3) derived during SMFT in detecting chronic training effects in neuromuscular function such as improved running efficiency (enhanced muscle–tendon unit recoil) is unknown.

#### Considerations

Most studies adopting mechanical outcome measures to denote acute neuromuscular effects have administered SMFT characterized by intermittent high-intensity bursts. These outcome measures are often sampled from the constant running velocity phases of the SMFT and using the vertical accelerometer component [52, 68, 167], perhaps owing to an enhanced association with vertical stiffness [52, 68]. Typically, such techniques have demonstrated an inferior degree of reliability (CV: 6.7–17.5%, standardized TE: small to moderate [49, 52, 68]) versus maximal and non-running-based assessments of neuromuscular function (jump and force indices; CV: 2.9–6.1%, standardized TE: small [49, 65, 171]). One study [65] using a continuous-fixed SMFT and lower running intensity (mean velocity of 12 km·h^−1^ compared to 18–22.5 km·h^−1^) reported lower measurement noise (CV: 2.1–8.0%, standardized TE rated as small). Additionally, the changes found in AL_V_ (%) [decreased] and AL_ML_ (AU) [increased] 24 h after a strenuous soccer training session were greater than smallest worthwhile change (signal-to-noise ratio >  ± 1) [167]. Although these findings indicate enhanced reliability and sensitivity for SMFT involving lower running speeds in a more continuous manner, this is based on only one study and the utility of different SMFT protocols has not yet been compared in the available literature, and barriers to implementation should also be considered. Similarly, the questionable reliability of locomotor outputs recorded during intermittent-variable SMFT in the form of SSG (total distance CV: 2.3–11.7%, high-speed thresholds CV: 8.1–83.0% [70, 72, 94, 98, 156, 157], small to moderate in magnitude [70, 156, 157], perhaps limits their utility to denote moderate-to-large effects only (i.e., larger CV may decrease the signal-to-noise ratio).

Although studies have suggested that changes in AL variables may reflect effects on lower limb stiffness [162, 172, 173], few have directly assessed stiffness [52, 174]. Moreover, these studies have typically used MEMS mounted between the scapulae, which may be influenced by upper-body kinematics during running or dampening of ground-contact vibrations [172, 175]. Whilst unit placement may have limited the impact under standardized conditions, intermittent-variable SMFT may be more susceptible to positioning noise as changes in orientation of the MEMS devices are not considered in the quantification of accelerometer metrics. In addition to positioning, users should be cautious of other extraneous factors such as movement artifacts within the device harness, running surface (e.g., ground stiffness), and footwear [158].

Future work should address the overall convergent validity of MEMS-derived data to obtain an accurate estimation of running strategy characteristics such as vertical stiffness. It is also necessary to investigate the theoretical framework for the sensitivity of these measures and their potential mechanisms (i.e., with respect to human tissue and gait mechanics). Furthermore, more research is required to examine whether protocol characteristics (e.g., exercise regimen, running intensity) and unit placement (e.g., center of mass, foot-mounted MEMS unit) can enhance measurement properties, and therefore facilitate inferences regarding lower limb stiffness and ultimately neuromuscular fatigue or efficiency.

## Summary and Conclusions

Our review provides an overview of the literature regarding SMFT in team sports, including the development of the SMFT definition, protocol categorization, and a systematic audit of protocols and outcome measures. We also provide a narrative synthesis of the applications of SMFT within the training continuum of sport teams and future research directions (outlined in Table 2). In summary, SMFT have the potential to serve as time-efficient, non-exhaustive, and feasible standardized tests that can be administered to a group of athletes simultaneously as a part of the warm-up and using specific drill(s) during the training session. Multivariate outcome measures such cardiorespiratory/metabolic (e.g., HR-derived indices), subjective (e.g., RPE), and mechanical (GPS and MEMS-derived data) can be collected simultaneously, and in theory, provide a multifactorial evaluation for athlete monitoring in team sports. Collectively, the literature suggests that several outcome measures collected during and immediately post-SMFT can inform on an athlete’s physiological state. Heart rate-derived indices seem more appropriate to denote positive chronic training effects on endurance performance, whereas their role in detecting negative transient effects associated with variations in ANS function is questionable. Despite the lack of knowledge about the underlying mechanisms and the inconsistent findings between studies, their sensitivity appears to improve after days or over short periods that are characterized by substantial alterations in training stress, seemingly caused by an interaction between cardiac ANS status and plasma volume responses. Subjective outcome measures are less common in team-based sports and only global RPE have been adopted thus far. Although their validity and practicability have yet to be established, researchers have proposed their utility when measured concomitantly with other objective measures (e.g., HRex) as part of a multivariate monitoring system. Mechanical outcome measures are relatively novel and have been mostly investigated using intermittent-variable and intermittent-fixed protocols, whereby the former primarily involves GPS-derived kinematic variables (levels 1–2) to monitor exercise performance, while the latter includes response measures derived from inertial measurement units (level 3) to monitor lower limb neuromuscular function. Whilst monitoring locomotor outputs during standardized training drills is more feasible and has shown to provide valuable data on an athlete’s performance, practitioners should consider the large influence of various individual and contextual factors (e.g., technical/tactical level, motivation) that may undermine their interpretations. Accelerometery load parameters can provide sensitive indicators of acute changes in lower limb function and therefore neuromuscular fatigue and efficiency, albeit the overall validity of these outcome measures and the physiological mechanisms underpinning their changes have not yet been fully evaluated. Moreover, there is an absence of information on the use of all mechanical metrics (levels 1–3) to monitor chronic training effects. Finally, future research should also examine the methodical elements (e.g., protocol characteristics, collection, and analytical processes) related to SMFT to derive the most appropriate protocol to capture reliable, valid, and sensitive outcome measures that provide useful inferences regarding an athlete’s physiological state.

**Table 2:** Practical applications and future directions

**SMFT protocol**			
•	In team sports, which comprise a large number of individuals, it is more practical to prescribe external (duration and velocity) rather internal intensity (e.g., fixed HR response) parameters		
•	Whilst (theoretically) individualized prescription of SMFT intensities accounts for the potential variation of physiological capacities within team-sports squads, this may be less feasible given the inherent requirement for periodic fitness assessments during seasonal stages		
•	When HR-derived indices are monitored, notably HRex, we recommend the use of continuous (fixed, incremental) SMFT protocols, with a minimum dose of 3–4 min to attain more stable HR during data collection		
•	Most studies administer intermittent-fixed, high-intensity SMFT to denote transient effects in lower limb function. This could be because researchers consider that higher velocities are required to examine neuromuscular status. Whilst no studies have explicitly examined this query, we encourage team-sport researchers to test the utility of continuous lower intensity SMFT		
•	In the absence of strong evidence of regarding the optimal SMFT exercise intensity, we recommend the prescription of fixed intensities that serve the training purposes (e.g., integrated into the warm-up), without negatively impacting the subsequent session, and can be easily repeated over time (i.e., individual and resource constraints)		
•	Because of the notable influence of individual and contextual factors, practitioners should consider administering intermittent-variable protocols (e.g., SSG) in combination with more generic SMFT, rather than isolation. For example, continuous SMFT during the warm-up and SSG within the main parts of the session. This may provide insight into general vs sport-specific training effects		
•	When monitoring the internal response to intermittent-variable SMFT (e.g., SSG HRex), we recommend quantifying and, if possible, controlling for external intensity parameters. The latter should be sought through linear regression techniques, with ratios (e.g., internal:external) being avoided		
**HR-derived indices**			
•	HRex is the most viable SMFT outcome measure to evaluate an athlete’s physiological state, principally to monitor chronic aerobic-oriented training effects		
•	Considering that similar changes in HR-derived indices can infer positive and negative training effects, researchers and practitioners are advised to include more than a single-outcome measure (e.g., HRex and RPE) and interpret the trends observed in relation to training aims and context		
•	We recommend caution to practitioners when interpreting changes in cardiac parasympathetic outcome measures (e.g., HRR), given their direct link with exercise intensity (i.e., changes in HRex may influence HRR)		
**Subjective**			
•	RPE may provide a useful adjunct to objective SMFT outcome measures such as HRex or accelerometry-load measures for monitoring physiological state, but data must be collected in an appropriate and standardized manner (i.e., appropriate anchoring and education, correct scales, in isolation) to mitigate the influence of conscious bias and habitual ratings		
•	The practicality of collecting SMFT RPE in the originally intended manner from groups of team-sport athletes (~ 20–40) is logistically challenging. However, this may be feasible if SMFT is performed as a discreet session that is not integrated within training, when working with individuals (e.g., small group training, or the rehabilitation return-to-play phase), or if data collection procedures are made more accessible (e.g., mobile applications, human resources)		
•	Differential RPE (respiratory, muscular) may provide a more sensitive appraisal of perceived exertion in response to change in physiological state, but the utility of these measures requires further investigation		
**Mechanical**			
•	Monitoring locomotor outputs such as distance covered and high-intensity efforts during SSG displays a larger magnitude of measurement error and therefore may compromise their sensitivity. We suggest future work scrutinizing whether modifications in game constraints can reduce physical performance variability, or alternatively explore the utility of drill-based exercises such as passing drills		
•	MEMS devices used within SMFT may be a promising tool to monitor lower limb neuromuscular status (fatigue and efficiency). However, before confident inferences can be made, further research is necessary to address their validity as markers of changes in running mechanics. The technology and the associated analytical techniques used, as well as other aspects such as the SMFT properties, outcome measures collected, and unit placement should be also considered		

*HR* heart rate, *HRex* heart rate exercise, *HRR* heart rate recovery, *MEMS* micro-electrical mechanical systems, *RPE* ratings of perceived exertion, *SMFT* submaximal fitness tests, *SSG* small-sided games

## Supplementary Information

Below is the link to the electronic supplementary material.

Supplementary file1 (PDF 104 KB)

Supplementary file2 (PDF 338 KB)

Supplementary file3 (PDF 273 KB)

## Declarations

### Availability of data and material

Supplementary materials for this paper are also available on the Open Science Framework at https://osf.io/f9xwj/.
