# Supplementary material for: Submaximal Fitness Tests in Team Sports: A Theoretical Framework for Evaluating Physiological State
Source: Sports Med. 2022 Jul 11;52(11):2605–26. doi: 10.1007/s40279-022-01712-0 (PMC9584880; doi:10.1007/s40279-022-01712-0)
Supplement: Supplementary file 1 — Supplementary file1 (PDF 104 KB) [file 40279_2022_1712_MOESM1_ESM.pdf]

Name: A historical summary of common SMFT adopted in clinical settings

Article Title: Submaximal Fitness Tests in Team Sports: A Theoretical Framework for Evaluating Physiological State

Journal: Sports Medicine

Authors: Tzlil Shushan[1], Shaun J. McLaren[2,3], Martin Buchheit[4,5,6,7], Tannath J. Scott[8,9], Steve Barrett[10] and Ric Lovell[1]

[1] School of Health Sciences, Western Sydney University, Sydney, NSW, Australia

[2] Newcastle Falcons Rugby Club, Newcastle upon Tyne, UK

[3] Department of Sport and Exercise Sciences, Durham University, Durham, UK

[4] HIIT Science, Revelstoke, BC, Canada

[5] French National Institute of Sport (INSEP), Laboratory of Sport, Expertise and Performance (EA 7370), Paris, France

[6] Kitman Labs, Performance Research Intelligence Initiative, Dublin, Ireland

[7] Institute for Health and Sport, Victoria University, Melbourne, VIC, Australia

[8] Netball Australia, Victoria, Australia

[9] Carnegie Applied Rugby Research (CARR) centre, Institute for Sport, Physical Activity and Leisure,

[10] Department of Sport Science Innovation, Playermaker, London, United Kingdom

Corresponding Author:

Tzlil Shushan

Email: Tzlil21092@gmail.com

Table 1 A historical summary of common SMFT adopted in clinical settings

SMFT	Year	Activity	Description	Outcome Measures
The Bruce Physical Index Test (PFI) [13]	1949	Walking	A single-stage to a maximum of 10-minute at 0.77 $m \cdot s^{-1}$ and 10% grade	Duration, oxygen uptake difference, HRR
Aerobic Capacity Fitness Test [14]	1954	Cycling	6-minute at workload that elicits HR steady-state between 125–170 $beats \cdot min^{-1}$	HR
Cyclic Step Test [20]	1971	Cycling	Cycling in a supine position during 3 variable workloads of 0, 300 and 650 $kpm \cdot min^{-1}$	HR, ventilation
Modified Bruce Physical Index Test [24,25]	1971<br>1973	Walking/Running	Multi-stage bouts while grade (up to 10%) and speed (up to ~10 $km \cdot h^{-1}$) are progressively increased	Duration, oxygen uptake difference, HRR
Physical Work Capacity 170 (PCW170) [21]	1976	Cycling	Cycling during 3 incremental workloads until a workload that elicits $beats \cdot min^{-1}$ or 75% HR maximum	Power (W)
Self-Paced Walking Test (SPWT) [15]	1976	Walking	3 self-paced stages walking bouts of 250 meters	Time, speed, stride frequency and mean HR
12-Minute Walk Test (12-MWT) [17]	1976	Walking	12 minutes self-paced walking	Distance
6-Minute Walk Test (6-MWT) [16]	1985	Walking	6 minutes self-paced walking	Distance
1-Mile Track Walk Test (1-MTW/Rockport Fitness Test) [18]	1987	Walking	One mile walking. Requires minimum of 2 similar tests	Duration and HR
The YMCA Cycle Ergometer Submaximal [22]	1989	Cycling	Usually includes 3 stages cycling bouts up to a workload that elicits 85% of HR maximum	Power (W)
Single Stage Submaximal Treadmill Walking Test (SSTWT) [19]	1991	Walking	4-minute 3 fixed speed stages ranged between 1.2–2 $m \cdot s^{-1}$ at grades of 0, 5 and 10%	HR and RPE
Modified Shuttle Walking [23]	1992	Walking/Running	Modified shuttle test, including 10-meter shuttles and 12 levels between 0.5–2.37 $m \cdot s^{-1}$	HR and RPE

HR heart rate, *HRR* heart rate recovery, *RPE* rating of perceived exertion
