# Supplementary material for: Submaximal Fitness Tests in Team Sports: A Theoretical Framework for Evaluating Physiological State
Source: Sports Med. 2022 Jul 11;52(11):2605–26. doi: 10.1007/s40279-022-01712-0 (PMC9584880; doi:10.1007/s40279-022-01712-0)
Supplement: Supplementary file 2 — Supplementary file2 (PDF 338 KB) [file 40279_2022_1712_MOESM2_ESM.pdf]

Name: Methodology Summary

Article Title: Submaximal Fitness Tests in Team Sports: A Theoretical Framework for Evaluating Physiological State

Journal: Sports Medicine

Authors: Tzlil Shushan[1], Shaun J. McLaren[2,3], Martin Buchheit[4,5,6,7], Tannath J. Scott[8,9], Steve Barrett[10] and Ric Lovell[1]

[1] School of Health Sciences, Western Sydney University, Sydney, NSW, Australia

[2] Newcastle Falcons Rugby Club, Newcastle upon Tyne, UK

[3] Department of Sport and Exercise Sciences, Durham University, Durham, UK

[4] HIIT Science, Revelstoke, BC, Canada

[5] French National Institute of Sport (INSEP), Laboratory of Sport, Expertise and Performance (EA 7370), Paris, France

[6] Kitman Labs, Performance Research Intelligence Initiative, Dublin, Ireland

[7] Institute for Health and Sport, Victoria University, Melbourne, VIC, Australia

[8] Netball Australia, Victoria, Australia

[9] Carnegie Applied Rugby Research (CARR) centre, Institute for Sport, Physical Activity and Leisure,

[10] Department of Sport Science Innovation, Playermaker, London, United Kingdom

Corresponding Author:

Tzlil Shushan

Email: Tzlil21092@gmail.com

Research Questions

The methodology component of our review was executed in accordance with PRISMA guidelines (Preferred Reporting Items for Systematic Reviews and Meta-Analyses) [1]. We defined our research questions by the PICOS model [1]:

Population

Team-sports athletes (refer to criteria 4 of the inclusion–exclusion criteria; Table 2).

Intervention (exposure)

1) No exposure, 2) Exposure to acute, short-term or chronic training regimen, 3) Exposure to extreme environments (heat, altitude). 4) Training interventions integrated into the general program such as high-intensity interval training.

Comparators

Submaximal Fitness Tests (SMFT).

Outcomes

Athlete's cardiorespiratory/metabolic, subjective, mechanical measures or any combination of which collected during or soon after SMFT.

Study design

We accepted any:

a) test-retest or correlational study designs: b) observational with single/repeated measures designs with or without reporting training loads, training context or environment; and c) training intervention studies that incorporated pre and post measurements.

Search Strategy

The electronic databases MEDLINE, Scopus, and Web of Science were used at multiple occasions (started on January 23rd, 2020, and finalised on August, 2021), including two independent searching strategies and combination of relevant free text terms linked to the Boolean operator 'OR' (levels). The final search was created by merging all term levels using the 'AND' operator (Table 1). The search syntaxes of the searching strategy are presented in Table 3. Further searches of the relevant literature were also conducted using other databases and reference lists.

Table 1 Searching Strategy

Level	Keywords
Level 1	submaximal OR sub-maximal OR "sub maximal" OR standardised OR standardized
Level 2	exercise OR test OR drill
Level 3	fitness OR fatigue
Level 4	physiological OR metabolic OR psychological OR psychometric OR mechanical OR biomechanical
Level 5	response OR responses
Level 6	athletes OR players OR "team sport" OR "team sports" OR "team based sport"
**First strategy (1 AND 2 AND 3 AND 6) OR Second strategy (1 AND 2 AND 4 AND 5 AND 6)**	

Screening Study and Selection

To select the appropriate articles, the first author (TS) initially exported the electronic search results to the reference management software Endnote (version X9), and then to a Microsoft Excel spreadsheet for further evaluation (N = 2170 records). Duplicates records were identified and removed (N = 1155), and an assessment of the remaining studies was undertaken according to the inclusion–exclusion criteria, in a sequential manner (i.e., criteria 1-8; Table 2). Consequently, more studies (title; N = 672 and abstract; N = 255) were discarded, and overall 107 full text studies were reviewed. This stage included multiple meeting and discussions to finalise the inclusion criteria for the review (refer to Table 2), its structure and data analyses. Finally, we accepted 69 studies from our searches, with an additional 18 studies ascertained from reference lists of relevant review papers (Figure 1).

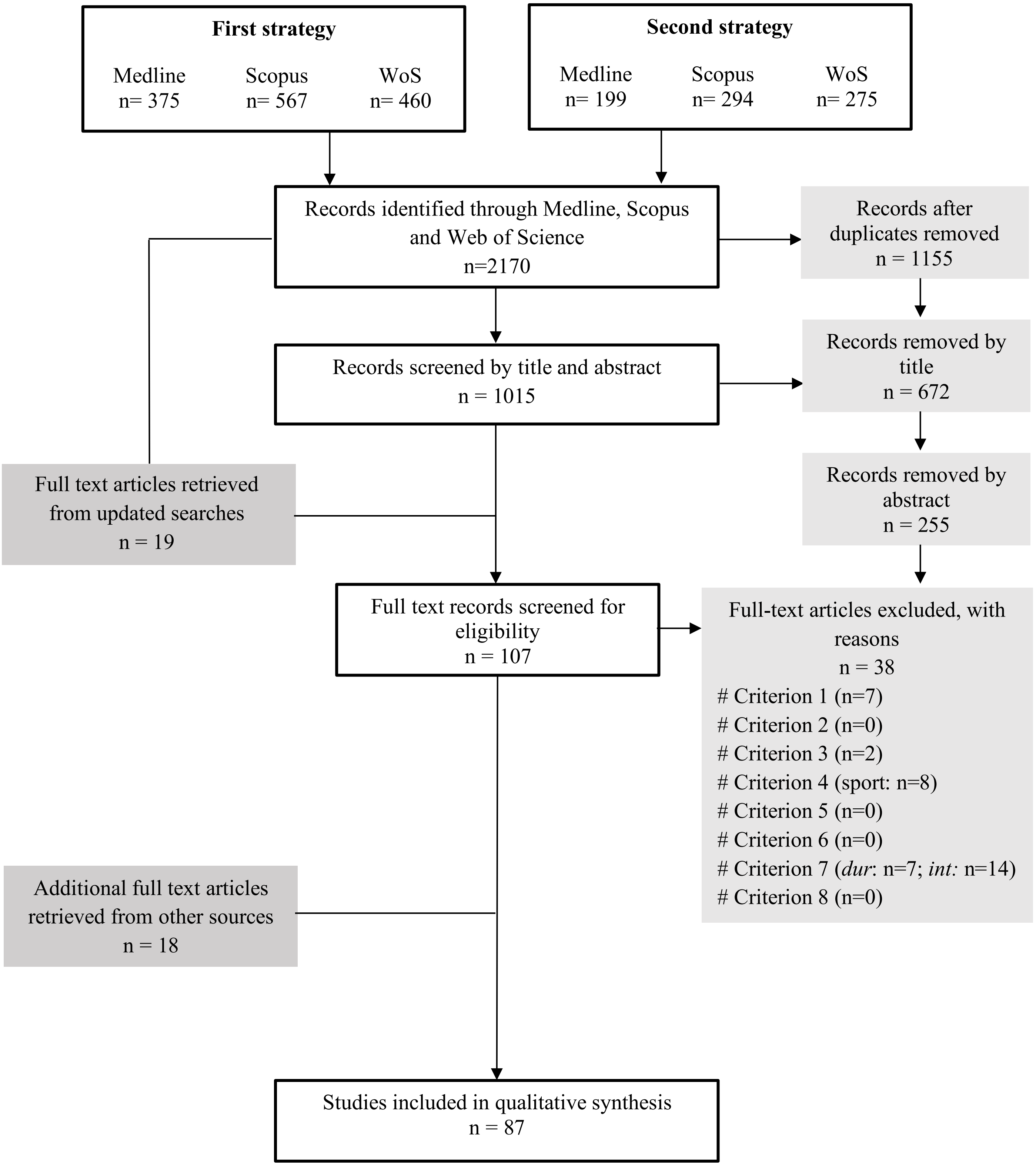

Fig. 1 PRISMA Flow Chart

Data Extraction and Analysis

The following data were independently extracted from each eligible study: 1) player characteristics: age, gender, sample size, sport and competition level; 2) methodological elements: study aims, design and training/testing settings; 3) SMFT characteristics: protocol, movement pattern, duration, intensity (external/internal parameters) and dimensions; and 4) outcome measures: cardiorespiratory/metabolic, perceptual and mechanical (the Supplementary File S3 illustrates the characteristics of our included studies).

Given the details attained from the included studies, we sought to construct the current review as follows: a systematic audit of the literature (including all eligible studies) was conducted to: i) generate SMFT definition and protocols taxonomy, ii) synthesise the independently described SMFT and their relevant outcome measure, and iii) evaluate the conceptual framework of using relevant outcome measures and the physiological mechanisms underpinning their changes. A narrative synthesis of SMFT within the team sports training effects continuum was conducted while focusing on studies investigating changes in relevant outcome measures, either in isolation or combination.

Table 2 Study Inclusion–Exclusion Criteria

Criteria	Inclusion	Description or reasons for exclusion
1	Original research published in peer-reviewed journal	Excluded non-investigation studies such as reviews, book chapters, abstracts, theses, opinion piece, surveys, letters to editor etc.
2	Studies published until August 2021	In accordance with our most updated searches, studies published from the inception up until August 2021 were considered for inclusion, including studies published in the *Epub ahead of print* format.
3	Available in English language	Full text is in English
4	Population	Team sport athletes with no restriction of age, level and gender.
5	Study Design	Test-retest designs<br>Studies examining the reliability of SMFT outcome measures (i.e., Criteria 8) and reporting absolute (intra-individual variability [typical error]) and/or relative (interclass correlation) estimates in controlled training settings (e.g., scheduling, training context).<br>Correlational designs<br>Studies examining the validity of SMFT outcome measures and reporting correlational statistic of the relationships between SMFT outcome measure and a criterion measure(s) (for example, the relationship between SMFT exercise heart rate and aerobic capacity test scores).<br>Observation with single/repeated measures<br>Including studies where usual training process was monitored and administered SMFT to infer athlete's physiological state (e.g., discriminate between individuals or evaluate training effects within individuals).<br>Training interventions with pre- and post-measurements<br>Including experimental trails where usual training process was coupled with an experimental intervention (e.g., high-intensity interval training) and SMFT were administered to infer athlete's training effects.
6	Settings	SMFT were administered either in laboratory, indoor/outdoor field formats or combination of two.
7	SMFT	Any SMFT including cycling, running, specific drills or games (refer to section 3.2 in the text: SMFT protocol taxonomy)
	Intensity	The intensity was non-exhausting (refer to section 3.1 in the text: SMFT definition)
	Duration	The duration was ≤15 minutes of *exercise* for all SMFT, while e*xercise* refers to the time when the outcome measures were monitored. Therefore, the duration of rest intervals during specific test protocols (e.g., recovery between sets during X bouts of incremental test or small-sided games) were not considered in the total duration, regardless if any outcome measure (e.g., heart rate recovery) was collected during this time.
8	Outcomes Measures	Cardiorespiratory/metabolic, subjective and/or mechanical

SMFT submaximal fitness tests

Table 3 Searching Strategy Syntaxes

MEDLINE (Strategy 1)

1 submaximal.mp. [mp=title, abstract, original title, name of substance word, subject heading word, floating sub-heading word, keyword heading word, organism supplementary concept word, protocol supplementary concept word, rare disease supplementary concept word, unique identifier, synonyms] (14701)

2 sub-maximal.mp. [mp=title, abstract, original title, name of substance word, subject heading word, floating sub-heading word, keyword heading word, organism supplementary concept word, protocol supplementary concept word, rare disease supplementary concept word, unique identifier, synonyms] (1223)

3 "sub maximal".mp. [mp=title, abstract, original title, name of substance word, subject heading word, floating sub-heading word, keyword heading word, organism supplementary concept word, protocol supplementary concept word, rare disease supplementary concept word, unique identifier, synonyms] (1223)

4 standardised.mp. [mp=title, abstract, original title, name of substance word, subject heading word, floating sub-heading word, keyword heading word, organism supplementary concept word, protocol supplementary concept word, rare disease supplementary concept word, unique identifier, synonyms] (33106)

5 standardized.mp. [mp=title, abstract, original title, name of substance word, subject heading word, floating sub-heading word, keyword heading word, organism supplementary concept word, protocol supplementary concept word, rare disease supplementary concept word, unique identifier, synonyms] (198666)

6 1 or 2 or 3 or 4 or 5 (246752)

7 exercise.mp. [mp=title, abstract, original title, name of substance word, subject heading word, floating sub-heading word, keyword heading word, organism supplementary concept word, protocol supplementary concept word, rare disease supplementary concept word, unique identifier, synonyms] (340290)

8 test.mp. [mp=title, abstract, original title, name of substance word, subject heading word, floating sub-heading word, keyword heading word, organism supplementary concept word, protocol supplementary concept word, rare disease supplementary concept word, unique identifier, synonyms] (1551077)

9 drill.mp. [mp=title, abstract, original title, name of substance word, subject heading word, floating sub-heading word, keyword heading word, organism supplementary concept word, protocol supplementary concept word, rare disease supplementary concept word, unique identifier, synonyms] (6662)

10 7 or 8 or 9 (1797753)

11 fitness.mp. [mp=title, abstract, original title, name of substance word, subject heading word, floating sub-heading word, keyword heading word, organism supplementary concept word, protocol supplementary concept word, rare disease supplementary concept word, unique identifier, synonyms] (87734)

12 fatigue.mp. [mp=title, abstract, original title, name of substance word, subject heading word, floating sub-heading word, keyword heading word, organism supplementary concept word, protocol supplementary concept word, rare disease supplementary concept word, unique identifier, synonyms] (103599)

13 11 or 12 (189408)

14 athletes.mp. [mp=title, abstract, original title, name of substance word, subject heading word, floating sub-heading word, keyword heading word, organism supplementary concept word, protocol supplementary concept word, rare disease supplementary concept word, unique identifier, synonyms] (48284)

15 players.mp. [mp=title, abstract, original title, name of substance word, subject heading word, floating sub-heading word, keyword heading word, organism supplementary concept word, protocol supplementary concept word, rare disease supplementary concept word, unique identifier, synonyms] (39760)

16 "team sport".mp. [mp=title, abstract, original title, name of substance word, subject heading word, floating sub-heading word, keyword heading word, organism supplementary concept word, protocol supplementary concept word, rare disease supplementary concept word, unique identifier, synonyms] (1082)

17 "team sports".mp. [mp=title, abstract, original title, name of substance word, subject heading word, floating sub-heading word, keyword heading word, organism supplementary concept word, protocol supplementary concept word, rare disease supplementary concept word, unique identifier, synonyms] (1346)

18 "team based sport".mp. [mp=title, abstract, original title, name of substance word, subject heading word, floating sub-heading word, keyword heading word, organism supplementary concept word, protocol supplementary concept word, rare disease supplementary concept word, unique identifier, synonyms] (7)

19 14 or 15 or 16 or 17 or 18 (81830)

20 6 and 10 and 13 and 19 (375)

MEDLINE (Strategy 2)

1 submaximal.mp. [mp=title, abstract, original title, name of substance word, subject heading word, floating sub-heading word, keyword heading word, organism supplementary concept word, protocol supplementary concept word, rare disease supplementary concept word, unique identifier, synonyms] (14701)

2 sub-maximal.mp. [mp=title, abstract, original title, name of substance word, subject heading word, floating sub-heading word, keyword heading word, organism supplementary concept word, protocol supplementary concept word, rare disease supplementary concept word, unique identifier, synonyms] (1223)

3 "sub maximal".mp. [mp=title, abstract, original title, name of substance word, subject heading word, floating sub-heading word, keyword heading word, organism supplementary concept word, protocol supplementary concept word, rare disease supplementary concept word, unique identifier, synonyms] (1223)

4 standardised.mp. [mp=title, abstract, original title, name of substance word, subject heading word, floating sub-heading word, keyword heading word, organism supplementary concept word, protocol supplementary concept word, rare disease supplementary concept word, unique identifier, synonyms] (33106)

5 standardized.mp. [mp=title, abstract, original title, name of substance word, subject heading word, floating sub-heading word, keyword heading word, organism supplementary concept word, protocol supplementary concept word, rare disease supplementary concept word, unique identifier, synonyms] (198666)

6 1 or 2 or 3 or 4 or 5 (246752)

7 exercise.mp. [mp=title, abstract, original title, name of substance word, subject heading word, floating sub-heading word, keyword heading word, organism supplementary concept word, protocol supplementary concept word, rare disease supplementary concept word, unique identifier, synonyms] (340290)

8 test.mp. [mp=title, abstract, original title, name of substance word, subject heading word, floating sub-heading word, keyword heading word, organism supplementary concept word, protocol supplementary concept word, rare disease

supplementary concept word, unique identifier, synonyms] (1551077)
9 drill.mp. [mp=title, abstract, original title, name of substance word, subject heading word, floating sub-heading word, keyword heading word, organism supplementary concept word, protocol supplementary concept word, rare disease supplementary concept word, unique identifier, synonyms] (6662)
10 7 or 8 or 9 (1797753)
11 physiological.mp. [mp=title, abstract, original title, name of substance word, subject heading word, floating sub-heading word, keyword heading word, organism supplementary concept word, protocol supplementary concept word, rare disease supplementary concept word, unique identifier, synonyms] (763561)
12 metabolic.mp. [mp=title, abstract, original title, name of substance word, subject heading word, floating sub-heading word, keyword heading word, organism supplementary concept word, protocol supplementary concept word, rare disease supplementary concept word, unique identifier, synonyms] (549587)
13 psychological.mp. [mp=title, abstract, original title, name of substance word, subject heading word, floating sub-heading word, keyword heading word, organism supplementary concept word, protocol supplementary concept word, rare disease supplementary concept word, unique identifier, synonyms] (473066)
14 psychometric.mp. [mp=title, abstract, original title, name of substance word, subject heading word, floating sub-heading word, keyword heading word, organism supplementary concept word, protocol supplementary concept word, rare disease supplementary concept word, unique identifier, synonyms] (41658)
15 mechanical.mp. [mp=title, abstract, original title, name of substance word, subject heading word, floating sub-heading word, keyword heading word, organism supplementary concept word, protocol supplementary concept word, rare disease supplementary concept word, unique identifier, synonyms] (378553)
16 biomechanical.mp. [mp=title, abstract, original title, name of substance word, subject heading word, floating sub-heading word, keyword heading word, organism supplementary concept word, protocol supplementary concept word, rare disease supplementary concept word, unique identifier, synonyms] (136147)
17 11 or 12 or 13 or 14 or 15 or 16 (2183069)
18 response.mp. [mp=title, abstract, original title, name of substance word, subject heading word, floating sub-heading word, keyword heading word, organism supplementary concept word, protocol supplementary concept word, rare disease supplementary concept word, unique identifier, synonyms] (2391179)
19 responses.mp. [mp=title, abstract, original title, name of substance word, subject heading word, floating sub-heading word, keyword heading word, organism supplementary concept word, protocol supplementary concept word, rare disease supplementary concept word, unique identifier, synonyms] (898430)
20 18 or 19 (2905304)
21 athletes.mp. [mp=title, abstract, original title, name of substance word, subject heading word, floating sub-heading word, keyword heading word, organism supplementary concept word, protocol supplementary concept word, rare disease supplementary concept word, unique identifier, synonyms] (48284)
22 players.mp. [mp=title, abstract, original title, name of substance word, subject heading word, floating sub-heading word, keyword heading word, organism supplementary concept word, protocol supplementary concept word, rare disease supplementary concept word, unique identifier, synonyms] (39760)
23 "team sport".mp. [mp=title, abstract, original title, name of substance word, subject heading word, floating sub-heading word, keyword heading word, organism supplementary concept word, protocol supplementary concept word, rare disease supplementary concept word, unique identifier, synonyms] (1082)
24 "team sports".mp. [mp=title, abstract, original title, name of substance word, subject heading word, floating sub-heading word, keyword heading word, organism supplementary concept word, protocol supplementary concept word, rare disease supplementary concept word, unique identifier, synonyms] (1346)
25 "team based sport".mp. [mp=title, abstract, original title, name of substance word, subject heading word, floating sub-heading word, keyword heading word, organism supplementary concept word, protocol supplementary concept word, rare disease supplementary concept word, unique identifier, synonyms] (7)
26 21 or 22 or 23 or 24 or 25 (81830)
27 6 and 10 and 17 and 20 and 26 (199)

Web of Science (Strategy 1)

TOPIC: (Submaximal OR sub - maximal OR "sub maximal" OR standardised OR standardized) AND TOPIC: (exercise OR test OR drill) AND TOPIC: (fitness OR fatigue) AND TOPIC: ("athletes" OR "players" OR "team sport" OR "team sports" OR "team based sport")

Web of Science (Strategy 2)

TOPIC: (Submaximal OR sub - maximal OR "sub maximal" OR standardised OR standardized) AND TOPIC: (exercise OR test OR drill) AND TOPIC: (physiological OR metabolic OR psychological OR psychometric OR mechanical OR biomechanical) AND TOPIC: (response OR responses) AND TOPIC: (athletes OR players OR "team sport" OR "team sports" OR "team based sport")

Scopus (Strategy 1)

(TITLE-ABS-KEY (submaximal OR sub-maximal OR "sub maximal" OR standardised OR standardized) AND TITLE-ABS-KEY (exercise OR test OR drill) AND TITLE-ABS-KEY (fitness OR fatigue) AND TITLE-ABS-KEY (athletes OR players OR "team sport" OR "team sports" OR "team based sport"))

Scopus (Strategy 2)

(TITLE-ABS-KEY (submaximal OR sub-maximal OR "sub maximal" OR standardised OR standardized) AND TITLE-ABS-KEY (exercise OR test OR drill) AND TITLE-ABS-KEY (physiological OR metabolic OR psychological OR psychometric OR mechanical OR biomechanical) AND TITLE-ABS-KEY (response OR responses) AND TITLE-ABS-KEY (athletes OR players OR "team sport" OR "team sports" OR "team based sport"))

Reference

1. Page MJ, Moher D, Bossuyt PM *et al.* PRISMA 2020 explanation and elaboration: updated guidance and exemplars for reporting systematic reviews. *BMJ* 2021:n160; https://doi.org/10.1136/bmj.n160.
