# Supplementary material for: Submaximal Fitness Tests in Team Sports: A Theoretical Framework for Evaluating Physiological State
Source: Sports Med. 2022 Jul 11;52(11):2605–26. doi: 10.1007/s40279-022-01712-0 (PMC9584880; doi:10.1007/s40279-022-01712-0)
Supplement: Supplementary file 3 — Supplementary file3 (PDF 273 KB) [file 40279_2022_1712_MOESM3_ESM.pdf]

Name: Characteristics of Included Studies

Article Title: Submaximal Fitness Tests in Team Sports: A Theoretical Framework for Evaluating Physiological State

Journal: Sports Medicine

Authors: Tzlil Shushan[1], Shaun J. McLaren[2,3], Martin Buchheit[4,5,6,7], Tannath J. Scott[8,9], Steve Barrett[10] and Ric Lovell[1]

[1] School of Health Sciences, Western Sydney University, Sydney, NSW, Australia

[2] Newcastle Falcons Rugby Club, Newcastle upon Tyne, UK

[3] Department of Sport and Exercise Sciences, Durham University, Durham, UK

[4] HIIT Science, Revelstoke, BC, Canada

[5] French National Institute of Sport (INSEP), Laboratory of Sport, Expertise and Performance (EA 7370), Paris, France

[6] Kitman Labs, Performance Research Intelligence Initiative, Dublin, Ireland

[7] Institute for Health and Sport, Victoria University, Melbourne, VIC, Australia

[8] Netball Australia, Victoria, Australia

[9] Carnegie Applied Rugby Research (CARR) centre, Institute for Sport, Physical Activity and Leisure,

[10] Department of Sport Science Innovation, Playermaker, London, United Kingdom

Corresponding Author:

Tzlil Shushan

Email: Tzlil21092@gmail.com

Supplementary File S3: characteristics of the included studies

Study	Participants					Methodology			SMFT						Outcome Measure		
	Age	Sex	N	Sport	Level	Settings	Design	Obs/Int period	Protocol	Move. Patt.	Test	Dur.	Int.	Dimensions	Cardiorespiratory/ Metabolic	Perceptual	Mechanical
Altman et al. 2020 (83)	23.7 ± 3.9	M	97	Soccer	E, NE	Training	RM	LT	CI	L	-	12min	6–12km·h^{-1}	Treadmill	HRex [%]		
Aoki et al. 2017 (60)	27.8 ± 6.4	M	9	Basketball	E	Training	Pre-post	LT	CF	C	5'-5'	5'-5'min	10km·h^{-1}	40m shuttle	HRex [%]<br>HRR [*%d*]		
Bradley et al. 2011 (84)	NS	M	32–46	Soccer	E	Training	RM	LT	II	C	Yo-YoIE2	4/6min	17.5km·h^{-1}	20m shuttle	HRex [a]<br>HRex [%]		
Bradley et al. 2014 (6)	23.0 ± 2.0	F	28	Soccer	E	Training	Correlational<br>RM	LT	II	C	Yo-YoIE2	2/4min	16.5/17.5<br>km·h^{-1}	20m shuttle	HRex [%]		
Brink et al. 2010 (86)	17.0 ± 0.5	M	18	Soccer	E	Training	RM	LT	II	C	ISRT	9–12min	14–15km·h^{-1}	20m shuttle	HRex [a]		
Brink et al. 2012 (85)	17.0 ± 1.0	M	7	Soccer	E	Training	RM	LT	II	C	ISRT	9–12min	14–15km·h^{-1}	20m shuttle	HRex [a]		
Brink et al. 2013 (87)	17.0 ± 1.0	M	14	Soccer	E	Training	RM	LT	II	C	ISRT	9–12min	14–15km·h^{-1}	20m shuttle	HRex [a]		
Buchheit et al. 2010 (45)	13.9<br>15.7	M	18<br>15	Soccer	E	Camp	RM	AC/ST	CF	L	5'-5'	5'-5'min	9km·h^{-1}	Track	HRex [%]<br>HRR [*d*]<br>Ln rMSSD		
Buchheit et al. 2011a (69)	17.0 ± 1.0	M	12	Ice Hockey	E	-	Test-retest	-	II	C	30-15IFT	3/6min	$^{-}$12.7/15.2<br>km·h^{-1}	40m shuttle	HRex [a]		
Buchheit et al. 2013b (56)	16.0 ± 0.4<br>18.1 ± 1.0	M	13<br>15	Soccer	NS	Altitude	RM	ST	CF	C	Short shuttle	5min	9km·h^{-1}	20m shuttle	HRex [%]	RPE[CR10]	
Buchheit et al. 2013c (108)	16.0 ± 0.4<br>18.1 ± 1.0	M	20<br>19	Soccer	NS	Altitude	RM	ST	CF	C	Short shuttle	5min	9km·h^{-1}	20m shuttle	HRex [%]	RPE[CR10]	
Buchheit et al. 2012 (107)	15.1 ± 1.5	M	46	Soccer	NE	Training	RM	LT	CF	L	5'-5'	5'-5'min	9km·h^{-1}	Track	HRex [%]<br>HRR [*%d*]<br>HRR [*d*]<br>Ln rMSSD		
Buchheit et al. 2011b (57)	26.2 ± 5.0	M	15	Soccer	NE	Heat	RM	AC/ST	CF	C	5'-5'	5'-5'min	9km·h^{-1}	~80m shuttle	HRex [%]<br>HRR [*d*]<br>Ln SD1	RPE[CR10]	
Buchheit et al. 2013a (106)	21.9 ± 2.0	M	18	ARF	E	Heat	RM	AC/ST	CF<br><br>IV	C<br><br>MD	5'-5'<br><br>5 star drills	5'-5'min<br><br>4–5min	13km·h^{-1}<br><br>-	40m shuttle<br><br>15-25m	HRex [%]<br>Ln SD1		 <br>Level 1:<br>TD, HIR
Buchheit et al. 2015 (58)	24.3 ± 4.2	M	36	ARF	E	Break	Pre-post	ST	CF<br>IV	C<br>MD	Long shuttle<br>Handball	5min<br>8min	12km·h^{-1}<br>-	~80m shuttle<br>141m^2/player	HRex [NA]	RPE[NA]	 Level 1:<br>TD, HIR,<br>maxV<br>Level 2:<br>Acc ± 3m·s^{-2}
Buchheit et al. 2016 (55)	24.6 ± 5.3	M	12	Soccer	E	Heat	RM	AC/ST	CF<br>IF	C<br>L	Rectangle<br>HIR bursts	4min<br>4×12sec	12km·h^{-1}<br>~18km·h^{-1}	100*50m<br>~60m	HRex [NA]		Level 3:<br>vL/fL
Buchheit et al. 2018 (49)	17.0 ± 2.0	M	18	Soccer	E	Training	RM	AC	IF	L	HIR bursts	4*12sec	22–24km·h^{-1}	~60m			Level 3:<br>Stiffness (K)<br>vL/fL

																	Fpeak
Buchheit et al. 2008 (61)	15.6 ± 0.8	M	15	Handball	NE	HIT/RST	Pre-post	LT	CF	L	-	6min	60%V_{IFT}	Track	HRex [%]<br>HRR [*d*]<br>HRR [a]<br>HRR [*T*]<br>rMSSD<br>SDNN		
Buchheit et al. 2020 (11)	23.0 ± 3.0	M	19	Soccer	E	Training	Pre-Post/<br>RM	LT	CI	L	-	12min	8–12km·h^{-1}	Treadmill	HRex [%]		
Castanga et al. 2010 (67)	15.3 ± 0.6	M	14	Basketball	E	-	Correlational	-	CI	C	Short shuttle	3×4min	9/10/11km·h^{-1}	20m shuttle	HRex [%]<br>B[La]	RPE[CR10]	
Cornforth et al. 2014 (102)	20.0 ± 3.0	M	20–27	ARF	E	Training	RM	LT	CI	C	Medium shuttle	4×2min	9.3/11.1/12.8/<br>14.6<br>km·h^{-1}	50m shuttle	HRex [a]<br>HRR[%*d*]		
Araújo et al. 2019 (105)	24.0 ± 4.0<br>24.0 ± 4.0<br>21.0 ± 4.0<br>25.0 ± 5.0	M<br>M<br>F<br>F	16<br>14<br>14–16<br>14–16	Soccer	E<br>NE<br>E<br>NE	-	Correlational	-	II	C	ISRT	2-9min	11–14km·h^{-1}	20m shuttle	HRex [%]<br>HRR [a]*		
de Freitas et al. 2015 (59)	24.9 ± 4.0	M	10	Futsal	E	Training	Pre-post	LT	CF	L	5’-5’	5min	9km·h^{-1}	20m rectangle	HRex [%]<br>HRex [a]<br>HRR [*d*]<br>Ln rMSSD		
									II	C	Yo-YoIR1/2	2–4min	13.5–17.5km·h^{-1}	20m shuttle	HRex [%]<br>HRex [a]		
Deprez et al. 2014 (109)	12.5 ± 0.6<br>14.0 ± 0.5<br>16.1 ± 0.6	M	9–27<br>8–26<br>4–19	Soccer	M	-	Test-retest	-	II	C	Yo-YoIR1	3/4/7min	14–15km·h^{-1}	20m shuttle	HRex [%]<br>HRR [%]*		
Deprez et al. 2015 (110)	13.9 ± 0.5<br>16.2 ± 0.6<br>18.1 ± 0.4	M	22<br>10<br>4	Soccer	E	-	Test-retest	-	II	C	Yo-YoIR1	6.5min	14.5km·h^{-1}	20m shuttle	HRex [%]<br>HRR [%]*		
Delisle-Houde et al. 2019 (74)	22.7 ± 1.3<br>19.9 ± 1.8	M<br>F	24<br>20	Ice Hockey	NE	Training	RM	LT	CF	L	Cycling	4min	75%VO_2 power	-	HRex [a]<br>HRR [a]<br>B[La]		
Dello Iacono et al. 2019 (70)	18.5 ± 0.6<br>18.7 ± 0.6	M	10<br>10	Soccer	E	-	Test-retest	-	IV	MD	Passing drill	12min	-	Half-two third pitch	HRex [%]	RPE[CR10]	Level 1:<br>TD, HIR, SR
Dillern et al. 2017 (75)	21.9 ± 3.5	M	17	Soccer	NS	Post-match	RM	AC	CF	L	-	10min	90%AT	Treadmill	HRex [a]<br>Oxygen uptake<br>RER<br>B[La]	RPE[6-20]	
Dubois et al. 2018 (90)	26.9 ± 1.9	M	14	Rugby Union	E	Training	RM	LT	CF	L	5’-5’	5’-5’min	10/11km·h^{-1}	Treadmill	HRex [a]<br>HRR [a]		
Dobbin et al. 2018 (76)	20.4 ± 1.2	M	17	Rugby	NE	-	Test-retest	-	II	C	Yo-YoIR1	1.5/2.5/4 min	13–14km·h^{-1}	20m shuttle	HRex [a]<br>Oxygen uptake<br>Ventilation<br>B[La]	RPE[6-20]	Level 3:<br>AL_{VM} (AU)
Dobbin et al. 2020 (88)	17.1 ± 1.0	M	31	Rugby	E	SIT	Pre-Post	ST	II	C	Yo-YoIR1	1.5/2.5/4 min	13–14km·h^{-1}	20m shuttle	HRex[a]<br>HRR[*d*]		Level 2:<br>Acc >3m·s^{-2}<br>MP<br>Level 3:<br>AL_{VM} (AU)
Doncaster et al. 2018 (89)	12.9 ± 0.7	M	8	Soccer	E	-	Test-retest	-	II	C	Yo-YoIR1	3/6min	14/14.5km·h^{-1}	20m shuttle	HRex [a]<br>HRex [%]<br>HRR [a]		

															HRR [%]		
Fanchini et al. 2014 (92)	17.0 ± 1.0	M	24	Soccer	E	Training	Test-retest<br>Correlational<br>Pre-post	LT	II	C	Yo-YoIR1	6min	14.5km·h^{-1}	20m shuttle	HRex[a]		
Fanchini et al. 2015 (91)	24.0 ± 6.0	M	11–13	Soccer	NE	Training	Pre-post	LT	II	C	Yo-YoIR1	6min	14.5km·h^{-1}	20m shuttle	HRex [a]<br>HRex [%]	RPE[CR100]	
Fitzpatrick et al. 2019a (65)	17.4 ± 0.5	M	15	Soccer	E		Test-retest	-	CF	C	Short shuttle	2–4min	12km·h^{-1}	20m shuttle			Level 3:<br>AL_{VM} (AU)<br>AL_{VM} (%)
Fitzpatrick et al. 2019b (167)	17.5 ± 0.5	M	12	Soccer	E	Training	Pre-post	AC	CF	C	Short shuttle	3min	12km·h^{-1}	20m shuttle			Level 3:<br>AL_{VM} (AU)<br>AL_{VM} (%)
Ferioli et al. 2018 (77)	25.6 ± 6.0<br>23.3 ± 4.7	M	14<br>18	Basketball	E<br>NE	Training	Pre-post	LT	CF	L	Mognomi's	6min	13.5km·h^{-1}	NS	B[La]		
Fox et al. 2017 (121)	19.0 ± 1.0	M	13	Soccer	E	Training	RM	LT	II	C	Yo-YoIR1	6min	14.5km·h^{-1}	20m shuttle	HRex<br>HRR [*d*]<br>HRR [*%d*]		
Francini et al. 2019 (111)	12.0-16.0	M	68	Soccer	NE	-	Correlational	-	CF	L	Mognomi's	6min	13.5km·h^{-1}	Track	HRex [%]<br>B[La]<br>H^+<br>HCO^3		
									II	C	Yo-YoIR1	6min	14.5km·h^{-1}	20m shuttle	HRex [%]		
Garret et al. 2019a (68)	22.5 ± 4.2<br>22.3 ± 2.9	M	12<br>11	ARF	E<br>NE	Post-match	RM	AC	IF	L	HIR bursts	8sec	~22.5km·h^{-1}	50m			Level 3:<br>AL_{VM} (AU)
Garret et al. 2019b (168)	22.5 ± 4.2<br>22.3 ± 2.9	M	12<br>11	ARF	E<br>NE	-	Test-retest	-	IF	L	HIR bursts	8sec	~22.5km·h^{-1}	50m			Level 3:<br>AL_{VM} (AU)
Garret et al. 2021 (169)	18.3 ± 0.5	M	20	Soccer	NE	Post-match	Pre-post	AC	IF	L	HIR bursts	10sec	~18km·h^{-1}	50m			MaxV
Garvican et al. 2014 (93)	18.8 ± 1.0	M	12	Soccer	E	Altitude	RM	AC/ST	CF	C	Short shuttle	5min	11km·h^{-1}	20m shuttle	HRex [a]		
Haddad et al. 2013 (50)	18.2 ± 0.5	M	17	Soccer	E	Training	RM	LT	CF	L	Track	10min	75% $HR_{RESERVE}$	-		RPE[CR10]	
Harry & Booysen 2020 (123)	23.0 ± 3.7	F	32	Field Hockey	NE	-	Correlational	-	II	C	Yo-YoIR1	6 min	14.5km·h^{-1}	20m shuttle	HRR [*d*]		
Hulin et al. 2019 (51)	24.1 ± 3.4	M	17–32	Rugby	E	Training	Test-retest<br>Pre-post	-<br>LT	II	C	Yo-YoIR1	4/6min	14/14.5km·h^{-1}	20m shuttle	HRex [%]		
Hulin et al. 2020 (118)	24.1 ± 4.0	M	45	Rugby	E	Training		LT	II	C	Yo-YoIR1	4 min	14km·h^{-1}	20m shuttle	HRex [%]		
Hulka et al. 2015 (94)	17.7 ± 1.5	25	25	Football	E	-	Test-retest	-	IV	MD	4V4 SSG	3×4min	-	80m^2/player	HRex [a]		Level 1:<br>TD
Hulse et al. 2013 (95)	U9-11<br>U12-14<br>U15-18	M	13<br>15<br>18	Soccer	E	-	Test-retest	-	II	C	MSFT	5.5min	10.5km·h^{-1}	20m shuttle	HRex [a]<br>HRR [a]		
Iaia et al. 2017 (78)	17.0 ± 1.0	M	19	Soccer	NE	HIT	Pre-post	LT	CF	L	Mognomi's	6min	13.5km·h^{-1}	Track	B[La]		
Ingebrigtsen et al. 2012 (96)	26.0 ± 7.0<br>20.0 ± 3.0	M	12<br>39	Soccer	E<br>NE	-	Correlational	-	II	C	Yo-YoIR1/2	2/4min	14–16km·h^{-1}	20m shuttle	HRex [a]<br>HRex [%]		
Ingebrigtsen et al. 2014 (122)	22.0 ± 0.5	M	10–57	Soccer	M	-	Test-retest<br>Correlational	-	II	C	Yo-YoIR1/2	2/4/6min	14–16.5km·h^{-1}	20m shuttle	HRex [NA]		
	26.0 ± 5.0	M	10	Soccer	E	Training	RM	LT	CF	C	Rectangle	4min	12km·h^{-1}	100*50m	HRex [NA]		

Lacome et al. 2018 (71)									IV		5V5-10V10 SSG	NS	-	117±65m^2/ player			Level 1: TD, HIR<br>Level 3: vL, fL, MechW
Lignell et al. 2018 (112)	28.0 ± 1.0	M	18	Ice Hockey	E	Post-match	Pre-post Correlational	AC	II	C	Yo-YoIR1	6min	14.5km·h^{-1}	20m shuttle	HRex [%]		
Leduc et al. 2019 (52)	21.0 ± 1.3	M	17	Rugby	NE	Training	RM	AC	IF	L	HIR bursts	12sec	~18km·h^{-1}	~60m			Level 3: RLI
Mohr & Krustrup 2014 (46)	25.8 ± 4.1	M	172	Soccer	NE	Training	RM Correlational	LT	II	C	Yo-YoIR1	6min	14.5km·h^{-1}	20m shuttle	HRex [%]		
Malone et al. 2017 (54)	24.3 ± 6.1	M	22	Gaelic Football	E	Camp	RM	AC/ST	CF	C	5’-5’	5’-5’min	13km·h^{-1}	40m shuttle	HRex [%]<br>HRR [*d*]		
									IV	MD	Touchdown	4min	-	150m^2/player			Level 1: TD, HIR, maxV
Meylan et al. 2021 (104)	27.0 ± 5.0	F	16	Soccer	E	Heat	RM	ST	CF	C	Medium shuttle	5min	12km·h^{-1}	40m shuttle	HRex[a]<br>HRR [*d*]	RPE	
									IV	MD	4V4 SSG	4×2min	-	140m^2/player	HRex[a]		TD<br>IMA
Moreira et al. 2021 (103)	19.0 ± 1.0	M	12	Soccer	E	Post-match	Pre-post	AC	CF	C	Medium shuttle	5min	10km·h^{-1}	40m shuttle	HRex[a]<br>HRR [*d*]		
Nakamura et al. 2017 (117)	24.5 ± 3.7<br>24.8 ± 5.6	M	17<br>25	Rugby Union	E	-	Performance test	-	II	C	Yo-YoIR1	2min	13.5km·h^{-1}	20m shuttle	HRex [%]		
Natera et al. 2019 (97)	28.1 ± 1.5	M	8	Rugby Union	NE	-	Test-retest	-	II	C	30-15IFT	-	13km·h^{-1}<br>25/50/75%V_{IFT}	40m shuttle	HRex [a]<br>HRex [RESERVE]<br>HRR [a]		
Nikolaidis et al. 2015 (155)	13.8 ± 0.6	F	28	Volleyball	NS	-	Correlational	-	CI	L	PCW170	3×3min	Up to 170 bpm	-			Power (W)
Owen et al. 2012 (72)	24.5 ± 3.5	M	15	Soccer	E	Training	Pre-post	LT	CI	L	-	3×3min	9/11/14 km·h^{-1}	Treadmill	Oxygen uptake<br>RER<br>HRex [a]		
Owen et al. 2017 (99)	17.8 ± 0.5	M	10	Soccer	E	-	Test-retest	-	II	C	Yo-YoIR1	6min		20m shuttle	HRex [a]<br>HRR [a]<br>HRR [%*d*]		
Owen et al. 2020 (98)	25.3 ± 3.1	M	23	Soccer	E	-	Test-retest Correlational	-	IV	MD	5V5 SSG	3×3min	-	62.5m^2/player	HRex [a]<br>HRex [%]	RPE[CR10]	Level 1: TD, HIR<br>Level 2: Acc, Dec, MP<br>Level 3: DSL
Pereira et al. 2019 (100)	11.5 ± 0.6	M	40	Soccer	NE	-	Correlational	-	CF	C	5’-5’	5’-5’min	9km·h^{-1}	20m rectangle	HRex [a]<br>HRR [*d*]<br>Ln rMSSD		
Prajapat et al. 2019 (125)	21.1 ± 2.1<br>21.4 ± 2.1<br>20.4 ± 1.6	M	20<br>18<br>17	Soccer<br>Basketball<br>Field Hockey	NE	-	Performance test	-	CI	L	-	-	Up to HRT	Treadmill	HRR [a]		

Rabbani et al. 2018a (66)	25.1 ± 4.0<br>26.7 ± 4.9	M	12–14	Soccer	E	-	Test-retest<br>Correlational	-	CF	C	Long shuttle	4min	12$km\cdot h^{-1}$	100m shuttle	HRex [a]<br>HRex [%]<br>HRR [*d*]<br>HRR [*%d*]<br>HRR [a]<br>HRR [%]	
Rabbani et al. 2018b (113)	26.1 ± 5.7	M	14	Soccer	E	Training	Pre-post	LT	CF	C	Long shuttle	4min	12$km\cdot h^{-1}$	100m shuttle	HRex [%]<br>HRR [*%d*]<br>HRR [%]	
Rago et al. 2020 (120)	27.8 ± 3.9	M	17	Soccer	E	Training	RM	LT	II	C	Yo-YoIR1	6:51min	14.5$km\cdot h^{-1}$	20m shuttle	HRex [%]	
Racinais et al. 2014 (132)	21.9 ± 2.0	M	18	ARF	E	Heat	RM	AC/ST	IV	MD	5 star drills	4-5min	-	15-25m		Level 1:<br>TD, HIR
Rampinini et al. 2021 (79)	25.4 ± 5.0	M	50	Soccer	E	Breat	Pre-post	LT	CF	L	Mognomi's	6min	13.5$km\cdot h^{-1}$	300m track	B[La]	
Reinhardt et al. 2020 (70)	23.5 ± 3.7	M	14	Soccer	NE	-	Correlational	-	IV	MD	4V4 SSG	6×1.5min	-	120m^2/player	HRex [%]<br>HRR [*s*]<br>B[La]	Level 1:<br>TD, HIR<br>Level 2:<br>Acc
Rowell et al. 2018 (72)	25.2 ± 5.5	M	21	Soccer	E	Training	RM	LT	IV	MD	5V5+5 SSG	4×3min	-	135m^2/player		Level 1:<br>TD, HIR<br>Level 3:<br>AL, AL_{VM},<br>AL_{2D}, AL_{SLOW}<br>(AU)
Ryan et al. 2019 (119)	24.6 ± 4.0	M	45	ARF	E	Training	Test-retest<br>RM	LT	CF	C	Long shuttle	5 min	12$km\cdot h^{-1}$	80m shuttle	HRex [%]<br>HRR [*d*]	
Scott et al. 2018 (12)	23.2 ± 3.4	M	22	Rugby	E	-	Test-retest	-	CF	C	Ind. shuttle	4min	75%1500m/<br>60%V_{IFT}	57-68m shuttle	HRex [%]<br>HRR [*%d*]	
Signorelli et al. 2012 (82)	25.0 ± 4.0	M	162	Soccer	E	-	Performance test	-	CI	L	Incremental run	5min	8–12$km\cdot h^{-1}$	Treadmill	HRex [a]<br>Oxygen uptake	
Starling et al. 2021 (124)	23.0 ± 2.0		22	Rugby	NE	Post-match	RM	ST	CI	C	Short shuttle	5min	8–12km·h^{-}	20m shuttle	HRR [*d*]	
Thorpe et al. 2015 (53)	19.1 ± 0.6	M	10	Soccer	E	Training	RM	AC/ST	CF	L	5'-5' Cycling	5min	130W/85RPM	-	HRex [%]<br>HRR [*d*]<br>HRR [*%d*]<br>Ln rMSSD	
Thorpe et al. 2016 (47)	27.0 ± 5.1	M	29	Soccer	E	Training	RM	LT	CF	L	5'-5' Cycling	5min	130W/85RPM	-	HRex [a]<br>HRR [*d*]<br>HRR [*%d*]<br>Ln rMSSD	
Thorpe et al. 2017 (114)	19.1 ± 0.6	M	10	Soccer	E	Training	RM	AC/ST	CF	L	5'-5' Cycling	5min	130W/85RPM	-	HRex [%]<br>HRR [*d*]<br>HRR [*%d*]<br>Ln rMSSD	
Vachon et al. 2021 (126)	18.0 ± 1.0	M	13	Rugby Union	E	Training (taper)	Pre-post	ST	CF	L	5'-5' Cycling	5min	200W	-	HRex [NA]<br>HRR [*T*]	
Veugelers et al. 2016 (48)	23.0 ± 4.0	M	25–38	ARF	E	-	Test-retest<br>Correlational	-	II	C	Yo-YoIR2<br>Mod.<br>Yo-YoIR2	2–8min	16.5–17.5$km\cdot h^{-1}$<br>14.9–15.8$km\cdot h^{-1}$	20m shuttle	HRex [%]<br>HRR [*d*]	

Vigh-Larsen et al. 2019 (115)	23.5 ± 4.4<br>19.4 ± 2.9	M	245	Ice Hockey	M	-	Correlational	-	II	C	Yo-YoIR1	6min	$14.5km·h^{-1}$	20m shuttle	HRex [%]		
Younesi et al. 2021a (116)	27.2 ± 3.4	M	16	Soccer	E	-	Correlational	-	IV	MD	3V3 SSG	2×3min	-	$67.5m^2$/player	HRex [%]		Level 1: TD, HIR<br>Level 2: Acc $±2.2m·s^{-2}$
Younesi et al. 2021b (101)	28.1 ± 4.6	M	20	Soccer	E	-	Test-retest	-	IV	MD	3V3-6V6 SSG	3×3-6min	-	~$70m^2$/player	HRex [a]		
Younesi et al. 2021c (156)	28.1 ± 4.6	M	20	Soccer	E	-	Test-retest	-	IV	MD	3V3-6V6 SSG	3×3-6min	-	~$70m^2$/player			Level 1: TD, HIR<br>Level 2: Acc

N: sample size, **Obs**: observation, **Int:** intervention, **SMFT:** sub-maximal fitness test, **Dur:** durartion, **Int:** intensity, **M**: male**, F:** female, **E:** elite, **H+:** hydrogen ions, **HCO^3:** bicarbonate, **m:** meters, **min:** minute, **~:** approximately, **NE:** non-elite, **NS:** not specified, **RM:** repeated measures, **AC:** acute, **ST:** short-term, **LT:** long-term, **CF:** continuous-fixed**, CI:** continuous-incremental, **IF:** intermittent-fixed, **II:** intermittent-incremental, **IV:** intermittent-variable, **L:** linear, **C:** change of direction**, DB**: drill-based, **GB:** game-based, **$km·h^{-1}$:** kilometer per hour, **HIT:** high intensity interval training, **RST:** repeated sprint training, **30-15IFT**: 30-15 intermittent fitness test, **Yo-YoIR**1: yo-yo intermittent recovery test level 1, **Yo-YoIR2:** yo-yo intermittent recovery test level 2, **Yo-YoIE2:** yo-yo intermittent endurance level 2, **mod:** modified, **ISRT**: interval shuttle run test, **HIR**: high-intensity run, **SSG:** small sided game, **Ind:** individualised, **MSFT:** multi stage fitness test, **PCW 170**: physical work capacity test, **HRex:** exercise heart rate, **HRR:** heart rate recovery, **rMSSD:** square root of the root mean square of the sum of all differences between successive normal heartbeats heart rate variability, **Ln rMSSD:** log-transformed rMSSD**, Ln SD1:** natural log of standard deviation of successive R spikes measured from Poincaré plots, **SDNN:** standard deviation of mean interval differences between normal heartbeats, **B[La]:** lactate, **RER:** respiratory exchange ratio, **RPE:** rating of perceived exertion, **TD:** total distance, **maxV:** maximal velocity, **Mov Patt:** movement pattern, **vL:** velocity load, **fL**: force load **Acc:** acceleration, **IMA:** inertial movement analysis, **vL:** velocity load**, fL:** force load, **AL_{VM}:** accelerometry-load vector magnitude, **AL_{SLOW}**: component when the velocity is < 2m·s-1, **AL_{2D}**: individual vector component of AL excluding the vertical vector magnitude, **mechW:** mechanical work, **MP**: metabolic power, **DSL:** dynamic stress index, **RLI:** running load index, **SIT:** sprint interval training.
Obs/Int periods were classified as *acute* immediate, daily and up to one standard micro-cycle, *short-term* longer than one but less than four weeks, and *long-term* equal or more than four weeks.
*HRR include (*) were collected from maximal versions of the SMFT protocol.
